# Supplementary material for: MPAC: a computational framework for inferring pathway activities from multi-omic data
Source: bioRxiv. 2025 Jun 14:2024.06.15.599113. Originally published 2024 Jun 17. Preprint. [Version 3] doi: 10.1101/2024.06.15.599113 (PMC11212914; doi:10.1101/2024.06.15.599113)

bioRxiv preprint doi: https://doi.org/10.1101/2024.06.15.599113; this version posted June 14, 2025. The copyright holder for this preprint (which was not certified by peer review) is the author/funder, who has granted bioRxiv a license to display the preprint in perpetuity. It is made available under aCC-BY 4.0 International license.

# Supplementary Materials

## Supplementary figure legends

**Supplementary Figure 1. HPV- exploratory set patient samples grouped by GO term enrichment based on IPL (A), CNA (B), or RNA (C).** The selection threshold was lowered to ≥ 3 (**B**) or ≥ 60% (**C**) to include more GO terms to avoid bias.

**Supplementary Figure 2. HPV+ exploratory set patient samples grouped by GO term enrichment based on CNA (A) or RNA (B).** The selection threshold was lowered to ≥ 2 (**A**) or ≥ 60% (**B**) to include more GO terms to avoid bias.

**Supplementary Figure 3. HPV+ exploratory set patient samples grouped by MPAC's input RNA states. (A)** Clustering results by K-means under a pre-specified total number of groups ranging from two to six; (**B**) Clustering results by K-means with a pre-specified five groups; (**C–G**) Top significantly enriched GO terms in group I (**C**), II (**D**), III (**E**), IV (**F**), and V (**G**). The selection threshold was lowered to ≥ 95% (**F**), ≥70% (**G**), or ≥50% (**C–E**) in order to include more GO terms to avoid bias.

**Supplementary Figure 4. HPV+ exploratory set patient samples grouped by MPAC's input CNA states. (A)** Clustering results by K-means under a pre-specified total number of groups ranging from two to six; (**B**) Clustering results by K-means with a pre-specified three groups.

bioRxiv preprint doi: https://doi.org/10.1101/2024.06.15.599113; this version posted June 14, 2025. The copyright holder for this preprint (which was not certified by peer review) is the author/funder, who has granted bioRxiv a license to display the preprint in perpetuity. It is made available under aCC-BY 4.0 International license.

**Supplementary Figure 5. CNA, RNA, and pathway states of CD28 (A), CD86 (B), and LCP2 (C), as well as pathway states of their pathway network neighbors in the eleven group I patients.**

**Supplementary Figure 6. CNA, RNA, and pathway states of TYK2 (A) and IL12RB1 (B), as well as pathway states of their pathway network neighbors in the eleven group I patients.**

**Supplementary Figure 7. CNA, RNA, and pathway states of CD247 (A) and FASLG (B), as well as pathway states of their pathway network neighbors in the eleven group I patients.**

**Supplementary Figure 8. CNA, RNA, and pathway states of FASLG as well as pathway states of its pathway network neighbors in a group I patient TCGA-CR-7385.**

**Supplementary Figure 9. Overall survival of HPV+ exploratory set patient samples stratified by the inferred pathway levels (IPLs) of proteins from the same submodule (A) or individual protein (B).** Not all overall survival tests were statistically significant under a log-rank $p < 0.01$ cutoff.

**Supplementary Figure 10. Progression-free survival of HPV+ exploratory set patient samples stratified by the inferred pathway levels (IPLs) of all the seven proteins combined (A); proteins from the same submodule (B), or individual protein (C).** Not all progression-free survival tests were statistically significant under a log-rank $p < 0.01$ cutoff.

bioRxiv preprint doi: https://doi.org/10.1101/2024.06.15.599113; this version posted June 14, 2025. The copyright holder for this preprint (which was not certified by peer review) is the author/funder, who has granted bioRxiv a license to display the preprint in perpetuity. It is made available under aCC-BY 4.0 International license.

**Supplementary Figure 11. Immune cell compositions stratified by the inferred pathway levels (IPLs) of all the seven proteins combined for HPV+ exploratory (A) and validation (B) set.** Adjusted Wilcoxon test *p* on the cell composition difference between two groups of patient samples were shown for each cell type. Eosinophil was not found in either the exploratory or validation sets. T cell CD4+ naïve was not found in the validation set.

**Supplementary Figure 12. Overall survival of HPV+ validation set patient samples stratified by the inferred pathway levels (IPLs) of proteins from the same submodule (A) or individual protein (B).** Not all overall survival tests were statistically significant under a log-rank $p < 0.01$ cutoff.

**Supplementary Figure 13. Clustering patient samples and annotating patient groups by PARADIGM IPLs from Pan-Cancer Atlas.** K-means clustering applied to HPV+ exploratory (**A**) and validation (**C**) sets with a total number of groups ranging from two to five. Significantly overrepresented GO terms for groups of patients from HPV+ exploratory (**B**) and validation (**D**) sets. Note that one patient sample from HPV+ exploratory set does not have IPLs available in the Pan-Cancer Atlas's PARADIGM results, so only 70 of the 71 samples are shown in **A** and **B**.

**Supplementary Figure 14. Sub-sampling analysis by MPAC on HPV+ patient samples.** Patient groups and their significantly overrepresented GO terms by MPAC on randomly selected 10% (**A**), 30% (**B**), and 50% (**C**) of the 89 samples from HPV+ exploratory and validation set combined.

**Supplementary Figure 15. Exploratory and validation set resampling of MPAC on HPV+ patient samples.** The 71 exploratory set samples were divided into four groups with similar

bioRxiv preprint doi: https://doi.org/10.1101/2024.06.15.599113; this version posted June 14, 2025. The copyright holder for this preprint (which was not certified by peer review) is the author/funder, who has granted bioRxiv a license to display the preprint in perpetuity. It is made available under aCC-BY 4.0 International license.

sizes (18, 18, 18, and 17). The 18 validation set samples were used as the fifth group. The existing exploratory set and validation set was defined as split #1. Results for split #1 are shown in Figure 2 and Figure 4A. Patient groups and significantly overrepresented GO terms by MPAC are shown here for split #2 (**A** & **B**), #3 (**C** & **D**), #4 (**E** & **F**), and #5 (**G** & **H**).

**Supplementary Figure 16. Distribution of immune response patient samples under different numbers of permutations for HPV+ exploratory (A) and validation (B) set.** Immune response patient samples are defined as the eleven Group I exploratory set samples (Figure 2) and the six Group II validation set samples (Figure 4) from 100 permutations.

**Supplementary Figure 17. Impact of one, two, or three standard deviation thresholds on defining input RNA states for HPV+ validation set.** (**A**) Distributions of input RNA states; (**B**) Patient groups; (**C** & **D**) Significantly overrepresented GO terms in each patient group under one (**C**) or three (**D**) standard deviation threshold. Those under two standard deviations are shown in Figure 4A.

**Supplementary Figure 18. Comparison of the sizes of the largest and the second largest sub-network for HPV+ exploratory (A) and validation (B) set.** Sizes are represented by the total number of entities or the number of gene set genes that were used for downstream GO term overrepresentation analysis.

**Supplementary Figure 19. Comparison of the number of proteins that have their pathway states agreed with CNA and/or RNA-seq data.** All the 6,251 proteins that have CNA and RNA-seq data were stratified by whether their pathway states agree with neither of their CNA or RNA states (green), both CNA and RNA states (gray), only RNA but not CNA states (orange), or only CNA but not RNA states (blue).

bioRxiv preprint doi: https://doi.org/10.1101/2024.06.15.599113; this version posted June 14, 2025. The copyright holder for this preprint (which was not certified by peer review) is the author/funder, who has granted bioRxiv a license to display the preprint in perpetuity. It is made available under aCC-BY 4.0 International license.

**Supplementary Figure 20. MPAC cannot separate HPV+ and HPV- samples due to insufficient input pathway knowledge.** (**A**) MPAC grouping of patient samples from HPV+ (red) and HPV- (gray) exploratory set by their GO term overrepresentations; (**B**) Comparison of MPAC IPLs of two HPV subtype-specific proteins, CDKN2A and TP53, in patient samples from HPV+ (red) and HPV- (green) exploratory set. Wilcoxon *p*-values on the comparison are denoted.

**Supplementary Figure 21. Patient sample groups and significantly overrepresented GO terms for the TCGA cholangiocarcinoma cohort.**

**Supplementary Figure 22. Time (A) and memory (B) usage by MPAC's PARADIGM subroutine runs on real and permuted data from the 71 HPV+ exploratory set patient samples.** All the jobs were run via HTCondor on machines from the UW–Madison Center for High Throughput Computing.

bioRxiv preprint doi: https://doi.org/10.1101/2024.06.15.599113; this version posted June 14, 2025. The copyright holder for this preprint (which was not certified by peer review) is the author/funder, who has granted bioRxiv a license to display the preprint in perpetuity. It is made available under aCC-BY 4.0 International license.

## Supplementary Notes

### Supplementary Note 1. Pseudocode of MPAC.

```
FOR each sample:
  ## Step 1
  prepare CNA states from real data

  prepare RNA states from real data

  ## Step 2
     compute IPLs by PARADIGM using CNA and RNA states from real data as well as the input pathway definition

  ## Step 3
  REPEAT 100 times:
    randomly permute CNA and RNA states between genes

          compute IPLs by PARADIGM using CNA and RNA states from permuted data as well as the input pathway
          definition

  ## Step 4
  FOR each pathway entity:
    filter entity's IPL from real data by its 100 IPLs from permuted data

  ## Step 5
     based on the input pathway definition, select the largest sub-pathway with all of its entities with non-zero IPLs

  ## Step 6
  FOR each GO term in GMT file collections:
    do enrichment analysis using genes from the largest sub-pathway

cluster samples by their GO enrichment results

## Step 7
pick a sample group of interest

find submodules shared by the largest sub-pathways of all samples in this group

identify key pathway proteins from submodules

## Step 8
evaluate the association of key protein's IPLs with patients' clinical data
```

**Supplementary Note 2. Required number of permutations in MPAC.** To find out the number of permutations required for a valid result, we randomly selected 10, 20, and 50 permutations from the existing 100 permutations for each HPV+ sample. With them, we ran MPAC under the same protocol. We checked if the 11 and 6 immune response samples from the exploratory and validation set, respectively, were still grouped together and with no other samples added under a smaller number of permutations. For the exploratory set, when using 50 or 20 permutations, 9 or 10 of the 11 immune response samples continued to fall into the same group with at most 1 other sample added (Supplementary Figure 16A). When using 10 permutations, although 10

bioRxiv preprint doi: https://doi.org/10.1101/2024.06.15.599113; this version posted June 14, 2025. The copyright holder for this preprint (which was not certified by peer review) is the author/funder, who has granted bioRxiv a license to display the preprint in perpetuity. It is made available under aCC-BY 4.0 International license.

immune response samples were clustered together, 6 other samples came to the same group (Supplementary Figure 16A) and thus added more noise to the biological function of this group. For the validation set, the 6 immune response samples were spread almost evenly into different groups with at most 3 or 4 samples in a group with 2 or more other samples added by 50, 20, or 10 permutations (Supplementary Figure 16B). This analysis suggests that if a user has about 70 samples like the HPV+ exploratory set, 50 or 20 permutations would be ok. If a user has about 20 samples like the HPV+ validation set, 100 permutations would be required. In the MPAC package, we set the default to 100 and provide an option for users to adjust this number based on their sample sizes.

**Supplementary Note 3. Number of standard deviation threshold to define input RNA states.** The reason MPAC takes two standard deviations (SD) as the threshold is because it corresponds to the commonly used $p < 0.05$ cutoff. For any gene, MPAC extracts its expression levels from RNA-seq data of normal samples and fits them by a Gaussian distribution. Two-SD threshold on such a Gaussian distribution is at a $p = 0.05$ cutoff (three SD is at a $p = 0.01$ cutoff and one SD is at a $p = 0.32$ cutoff). To evaluate the impact by different SD, we tried 1, 2, and 3 SD on the 18 samples in the HPV+ validation set. We first checked their effects on the RNA states of all the genes. Those from 2 and 3 SD are similar to each other, especially for repressed and normal states (Supplementary Figure 17A), while those from 1 SD are largely different from those of 2 or 3 SD. Next, we compared the sample groups from different SD. Groups from 2 and 3 SD share good overlaps with three Group II samples in common (Supplementary Figure 17B), whereas groups from 1 SD are totally different from those from 2 and 3 SD. Such dissimilarity is further supported by the overrepresented pathways for each group. By 1 SD, its Group II has many GO terms related to cell cycle, Wnt signaling, etc, but very few related to immune response (Supplementary Figure 17C). In contrast, by 3 SD, its Group II has a large fraction of overrepresented GO terms related to immune response (Supplementary Figure 17D), which is similar to those by 2 SD (Figure 4A). Taken together, because of the commonly used $p < 0.05$ or $< 0.01$ cutoff, and because of the agreement on the RNA states and sample groups, we recommend users consider 2 or 3 SD to define input RNA states.

**Supplementary Note 4. The largest pathway sub-networks are much bigger than the second largest ones.** In MPAC, each sample has its own largest sub-network. The reason why MPAC only considers the largest sub-network is because the second largest sub-network is much smaller compared to the largest one. To illustrate this, we compared the sizes between the two from each sample in terms of two types of entities: (1) all entities in the sub-networks, which include proteins, complexes, families, etc; (2) only proteins that have their genes in the GO terms that were used for MPAC's enrichment analysis. In both HPV+ exploratory and validation set, the largest sub-networks are overwhelmingly larger than the second largest sub-networks for both all entities and GO terms genes (Supplementary Figure 18). The sub-network's 'stability' could be affected by a few factors, such as the number of permutations or the number of standard deviations to define input RNA states.

bioRxiv preprint doi: https://doi.org/10.1101/2024.06.15.599113; this version posted June 14, 2025. The copyright holder for this preprint (which was not certified by peer review) is the author/funder, who has granted bioRxiv a license to display the preprint in perpetuity. It is made available under aCC-BY 4.0 International license.

**Supplementary Note 5. CNA has a stronger impact than RNA-seq in determining protein pathway states.** MPAC integrates both CNA and RNA-seq data, but CNA's role is not evident from the key proteins shown earlier (Supplementary Figure 5-7). We extended the analysis to all the 6,251 pathway proteins with both CNA and RNA-seq data for all the 89 HPV+ exploratory and validation set samples. The comparison results are divided into the following four categories by whether a pathway state:

- Different from the one of CNA and RNA
- Same as the one of both CNA and RNA
- Same as the one of RNA, but different from the one of CNA
- Same as the one of CNA, but different from the one of RNA

For the majority of the 89 samples, the fraction of proteins that have pathway states only the same as CNA (blue bars in Supplementary Figure 19) is higher than those that have pathway states only the same as RNA (orange bars in Supplementary Figure 19), indicating that CNA has a stronger impact than RNA on determining a protein's pathway state overall. We also note that CNA does not have a stronger impact than RNA in every sample. 10 of the 89 samples have RNA with a stronger impact than CNA.

**Supplementary Note 6. MPAC cannot separate HPV+ and HPV- patients with insufficient input pathway information.** The reason we split HPV+ and HPV- before applying MPAC is because they are largely different in oncogenic pathways, tumor biology, and clinical treatment response. To test if MPAC can separate HPV+ and HPV-, we applied MPAC on the 393 samples from the HPV+ and HPV- exploratory sets. MPAC gives three groups and every group contains both HPV+ and HPV- samples (Supplementary Figure 20A), indicating that MPAC cannot separate HPV+ and HPV-. This is largely because the input pathways for MPAC have insufficient information on HPV. The pathways are from TCGA Pan-Cancer Atlas and are not designed specifically for HPV. They do not have any information on HPV protein E7 and very little on HPV protein E6. For example, it is well known that HPV E6 represses TP53 in HPV+, but MPAC's pathways do not contain such interaction. To illustrate further, we checked the MPAC IPLs of TP53 as well as another protein CDKN2A, which is known to have alterations predominantly only in HPV-. For both of them, their IPLs do not show a difference between HPV+ and HPV- (Supplementary Figure 20B). Despite there being a statistical difference of CDKN2A IPLs (Wilcoxon $p$=2.26x10$^{-5}$), CDKN2A and TP53's IPLs are within the same IPL ranges between HPV+ and HPV-, illustrating the impact of insufficient input pathway knowledge on key HPV-specific proteins and the difficulty on separating HPV+ and HPV-. The TCGA HNSCC study does not separate HPV+ and HPV- either using PARADIGM alone. In their paper's Supplementary Figure S7.11 (https://doi.org/10.1038/nature14129), HPV+ samples are mainly in subtype 3 (black), but this subtype also contains a substantial number of HPV- samples.

bioRxiv preprint doi: https://doi.org/10.1101/2024.06.15.599113; this version posted June 14, 2025. The copyright holder for this preprint (which was not certified by peer review) is the author/funder, who has granted bioRxiv a license to display the preprint in perpetuity. It is made available under aCC-BY 4.0 International license.

**Supplementary Note 7. Time and memory requirements for MPAC.** The major computational bottleneck for MPAC is running PARADIGM using the Pan-Cancer Atlas pathways on the large number of permuted data. For the 71 samples in HPV+ exploratory set, 100 permuted data for each sample resulted in 7,100 PARADIGM runs. This is the main reason why we utilized HTCondor from the Center for High Throughput Computing. Although most jobs on permuted data finished in 3 days, the longest one took about two weeks (Supplementary Figure 22A). In comparison, for jobs on real data, all of them finished in about four days (Supplementary Figure 22A). These jobs' memory usage is not heavy. Only 500 to 600 MB is needed for most of them for both real and permuted data (Supplementary Figure 22B). Jobs on permuted data may require longer time and more memory than on real data because permuted data likely have input omic states that are discordant with the input pathways and thus it is more computationally expensive to find optimal pathway states for both. Lastly, please note that, because of the nature of HTCondor, the aforementioned jobs were run on a heterogeneous resource (e.g., CPU speed, architecture).

**Supplementary Note 8. Changes in newer versions of the igraph R package affected the reproducibility of our results but not the main conclusions from our analyses.** Starting in igraph version 1.3, the Louvain method is no longer deterministic and different runs may generate different clustering results (https://github.com/igraph/rigraph/issues/539). To evaluate the impact of non-deterministic clustering, we executed three independent batches of 10,000 random Louvain clustering runs. We used the Adjusted Rand Index (ARI) to measure the difference between groups from these random runs with the original groups from igraph version 1.2.11. The median ARIs were 0.92, 1.00, and 0.78 for the HPV+ exploratory set, HPV+ validation set, and HPV- exploratory set, respectively, (HPV- validation set groups were not used in this analysis), indicating small variations in the patient cluster membership. The 11- and 6-patient immune groups in the HPV+ exploratory and validation set were largely unchanged (identical in >9,200 and >8,800 out of 10,000 runs, respectively). For the HPV- exploratory set, the top five grouping results in each batch have either 30- or 32-patient groups and both groups are enriched with immune response GO terms. In summary, despite the randomness introduced in igraph version 1.3, our findings are still maintained.

bioRxiv preprint doi: https://doi.org/10.1101/2024.06.15.599113; this version posted June 14, 2025. The copyright holder for this preprint (which was not certified by peer review) is the author/funder, who has granted bioRxiv a license to display the preprint in perpetuity. It is made available under aCC-BY 4.0 International license.

## Supplementary Tables

**Supplementary Table 1. Comparison of MPAC and PARADIGM features.** A check mark represents that feature is implemented and a cross mark indicates the lack of the feature.

| | MPAC | PARADIGM |
|---|---|---|
| Uses RNA-seq from normal samples to define RNA states | ✓ | ✗ |
| Uses prior pathway knowledge as input | ✓ | ✓ |
| Integrates CNA and RNA-seq data | ✓ | ✓ |
| Uses permuted input to identify randomly observed IPLs | ✓ | ✗ |
| Focuses on the largest sample-specific pathway sub-network | ✓ | ✗ |
| Clusters samples by their altered pathways | ✓ | ✗ |
| Defines the biological functions of sample groups | ✓ | ✗ |
| Identifies key proteins shared by samples in a group | ✓ | ✗ |
| Identifies the determinants of a protein's pathway state | ✓ | ✗ |

**Supplementary Table 2. Number of patient samples in each dataset.** HNSCC patient samples were stratified first by HPV subtypes and then by a random 80% and 20% split into exploratory and validation set to tune and test MPAC, respectively.

| | HPV+ | HPV- |
|---|---|---|
| **exploratory** | 71 | 322 |
| **validation** | 18 | 81 |

**Supplementary Table 3. Feature comparison of MPAC with other related software.** A check mark represents that feature is implemented and a cross mark indicates the lack of the feature.

| | MPAC | MOMA | OncoSig | COSMOS |
|---|---|---|---|---|
| Integrates multi-omic data | ✓ | ✓ | ✓ | ✓ |
| Utilizes prior pathway network knowledge as input | ✓ | ✗ | ✗[¶] | ✓ |
| Considers comprehensive molecular interactions at DNA, RNA, and protein level | ✓ | ✗[§] | ✗[§] | ✗[†] |
| Interprets result in terms of pathways | ✓ | ✓ | ✓ | ✓ |

bioRxiv preprint doi: https://doi.org/10.1101/2024.06.15.599113; this version posted June 14, 2025. The copyright holder for this preprint (which was not certified by peer review) is the author/funder, who has granted bioRxiv a license to display the preprint in perpetuity. It is made available under aCC-BY 4.0 International license.

| | | | | |
|---|---|---|---|---|
| Clusters samples by their pathway profiles and characterizes sample group by altered pathways | ✓ | ✓ | ✕ | ✕ |

¶ OncoSig takes a binary input on whether a protein is in a MSigDB gene set, but no interaction knowledge (i.e., pathway networks) can be used.

§ MOMA and OncoSig considers direct interactions on master regulators. Indirect interactions of proteins further downstream of master regulators are not included.

† COSMOS considers comprehensive molecular interactions over signaling pathways, gene regulations, and metabolic networks, but copy-number alteration (i.e., at DNA level) is not included.

bioRxiv preprint doi: https://doi.org/10.1101/2024.06.15.599113; this version posted June 14, 2025. The copyright holder for this preprint (which was not certified by peer review) is the author/funder, who has granted bioRxiv a license to display the preprint in perpetuity. It is made available under aCC-BY 4.0 International license.

**Supplementary Figure 1**

**A**

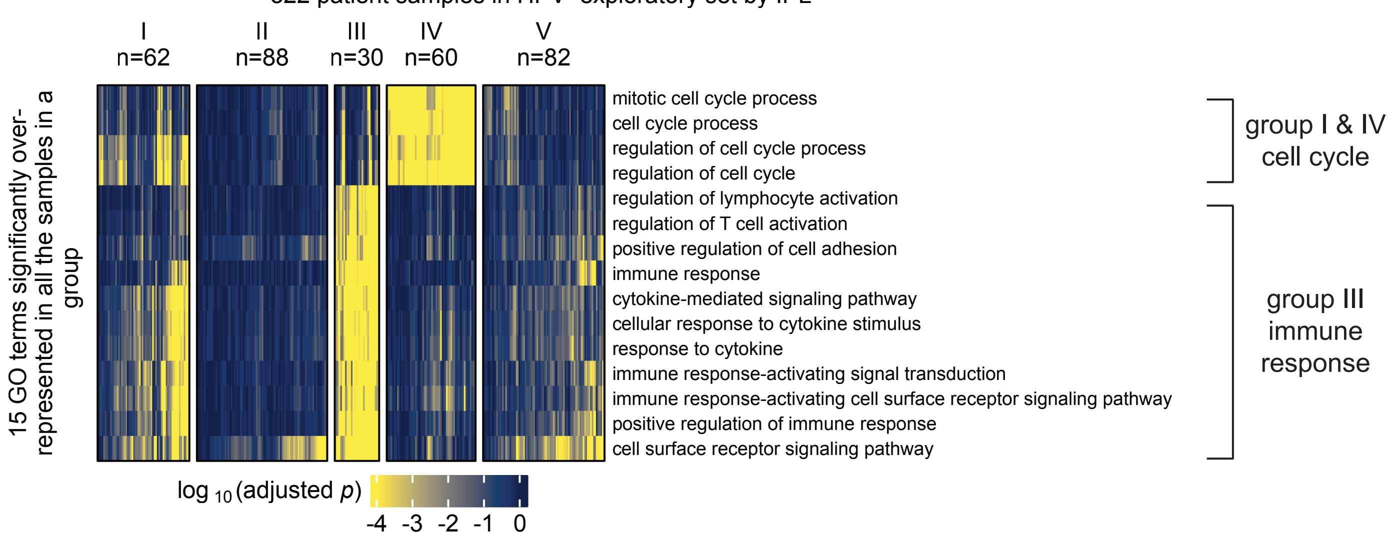


**B**

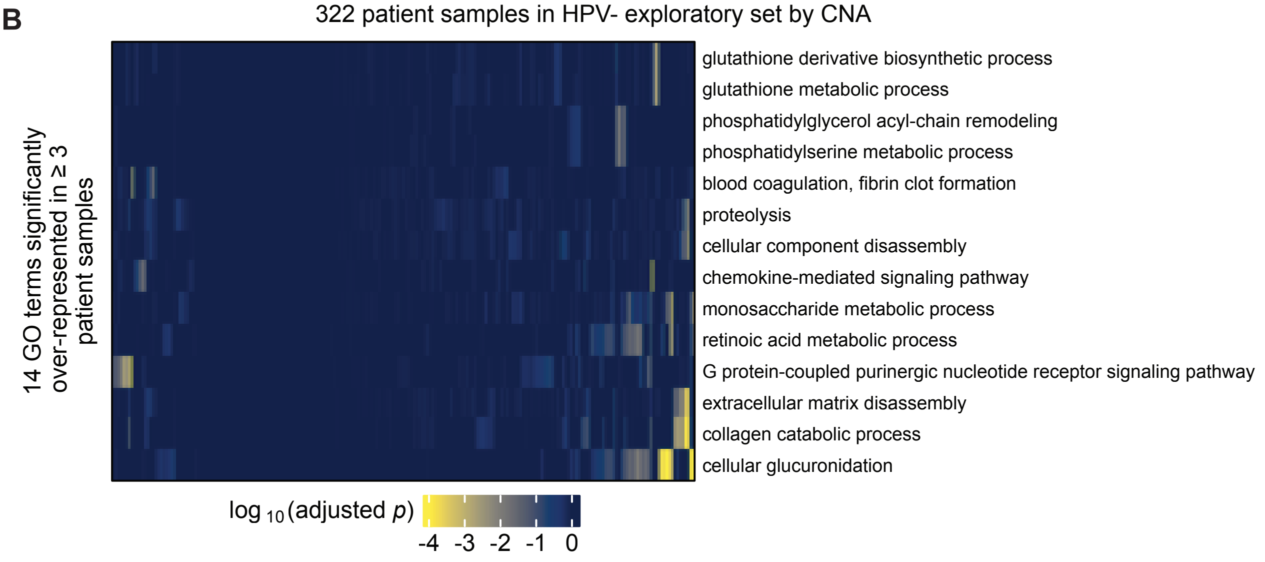


**C**

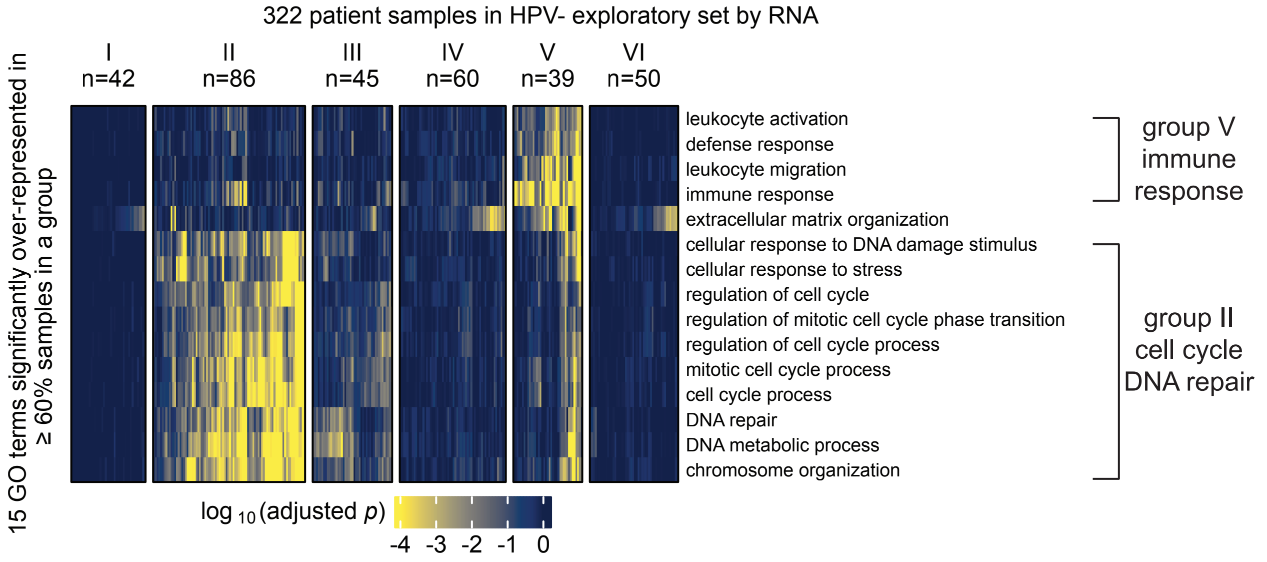

bioRxiv preprint doi: https://doi.org/10.1101/2024.06.15.599113; this version posted June 14, 2025. The copyright holder for this preprint (which was not certified by peer review) is the author/funder, who has granted bioRxiv a license to display the preprint in perpetuity. It is made available under aCC-BY 4.0 International license.

**Supplementary Figure 2**

**A**

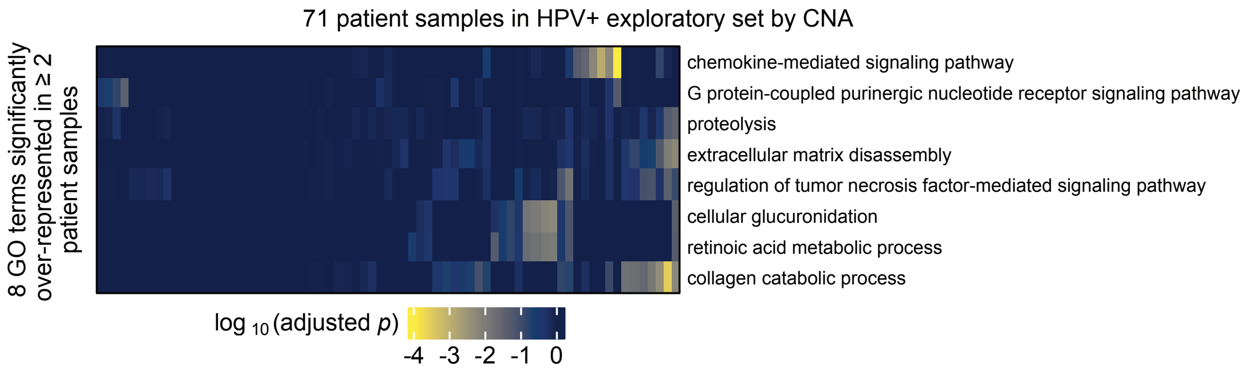


**B**

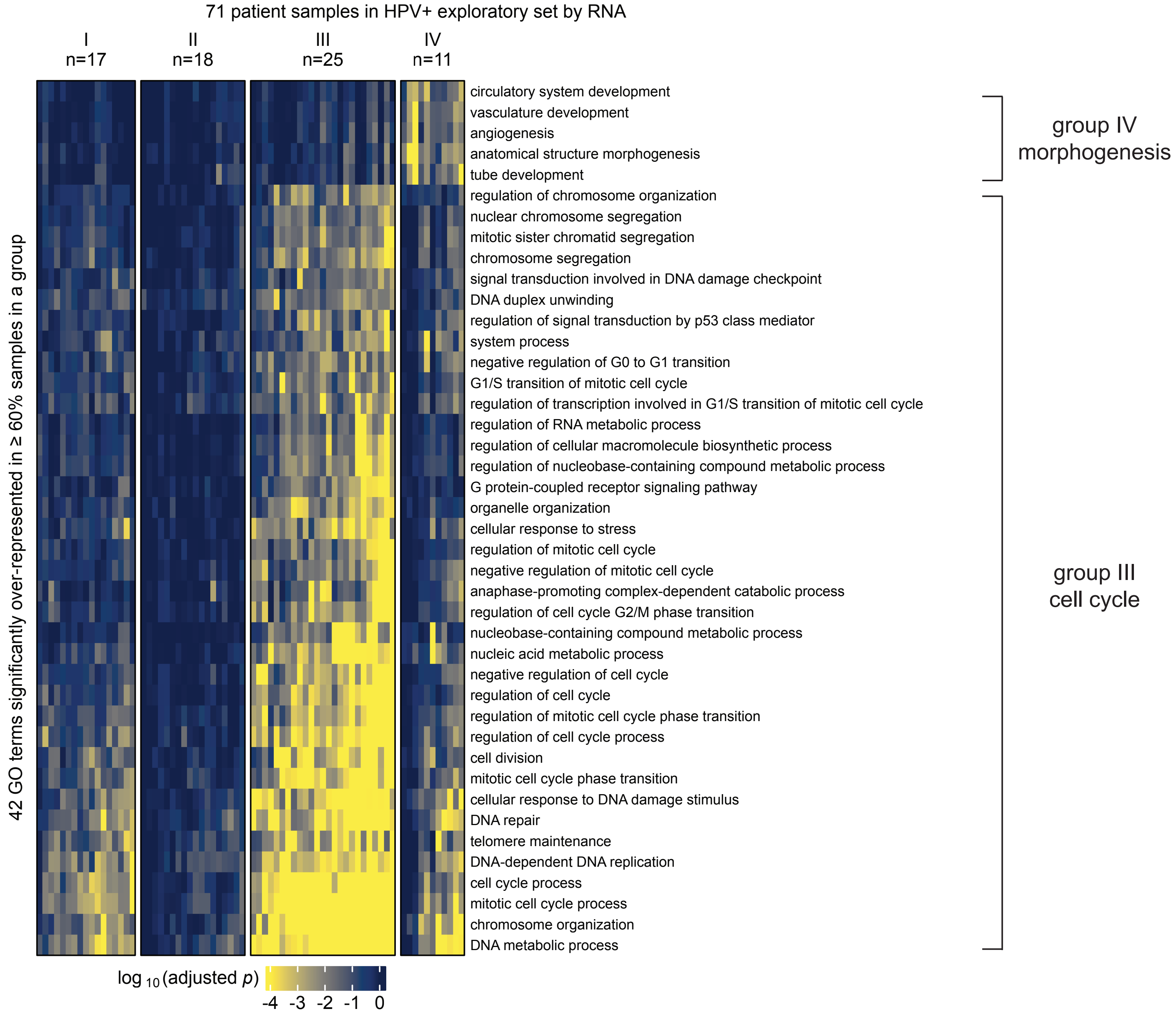

bioRxiv preprint doi: https://doi.org/10.1101/2024.06.15.599113; this version posted June 14, 2025. The copyright holder for this preprint (which was not certified by peer review) is the author/funder, who has granted bioRxiv a license to display the preprint in perpetuity. It is made available under aCC-BY 4.0 International license.

## Supplemenary Figure 3

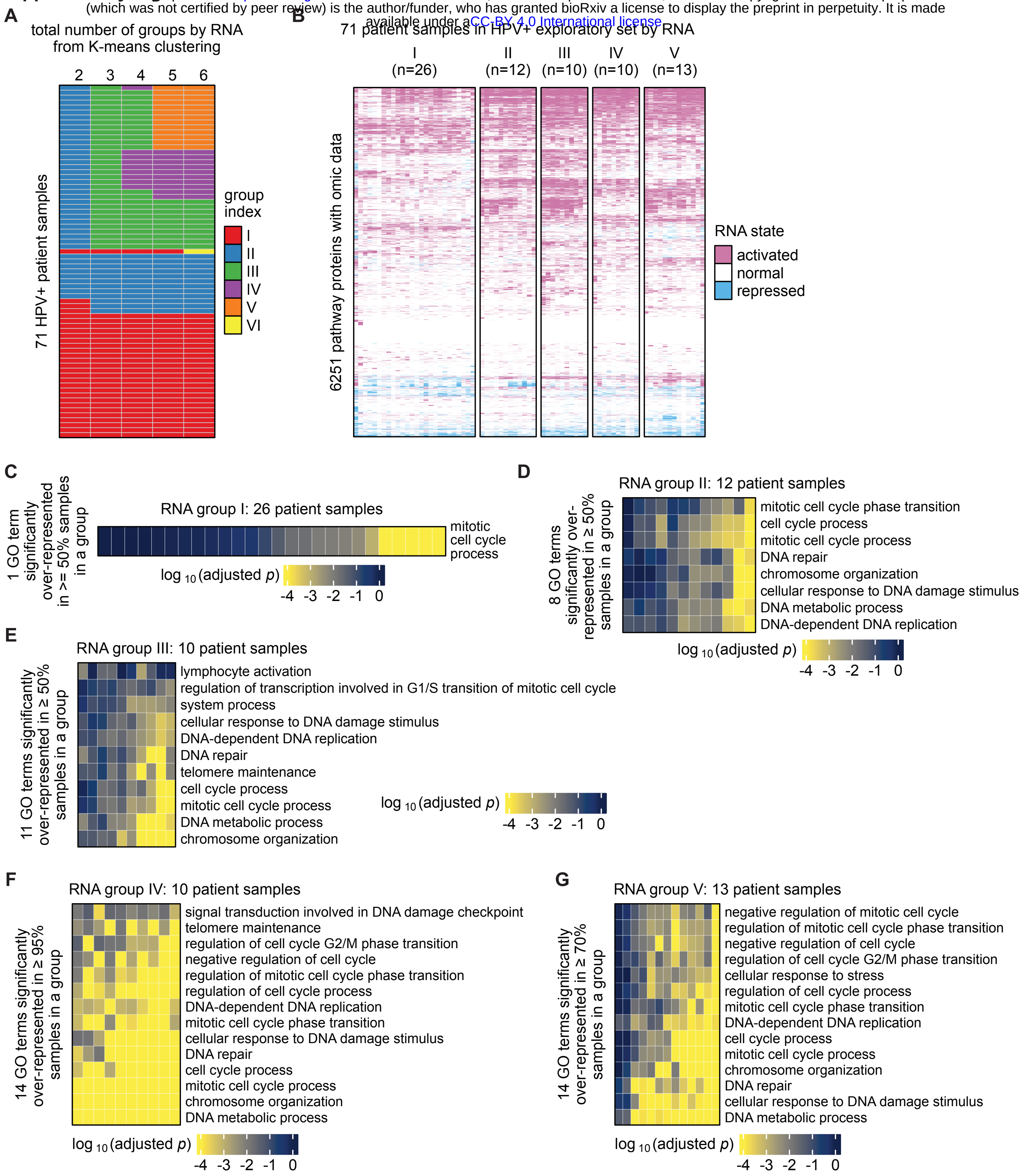

**Supplementary Figure 4**

bioRxiv preprint doi: https://doi.org/10.1101/2024.06.15.599113; this version posted June 14, 2025. The copyright holder for this preprint (which was not certified by peer review) is the author/funder, who has granted bioRxiv a license to display the preprint in perpetuity. It is made available under aCC-BY 4.0 International license.

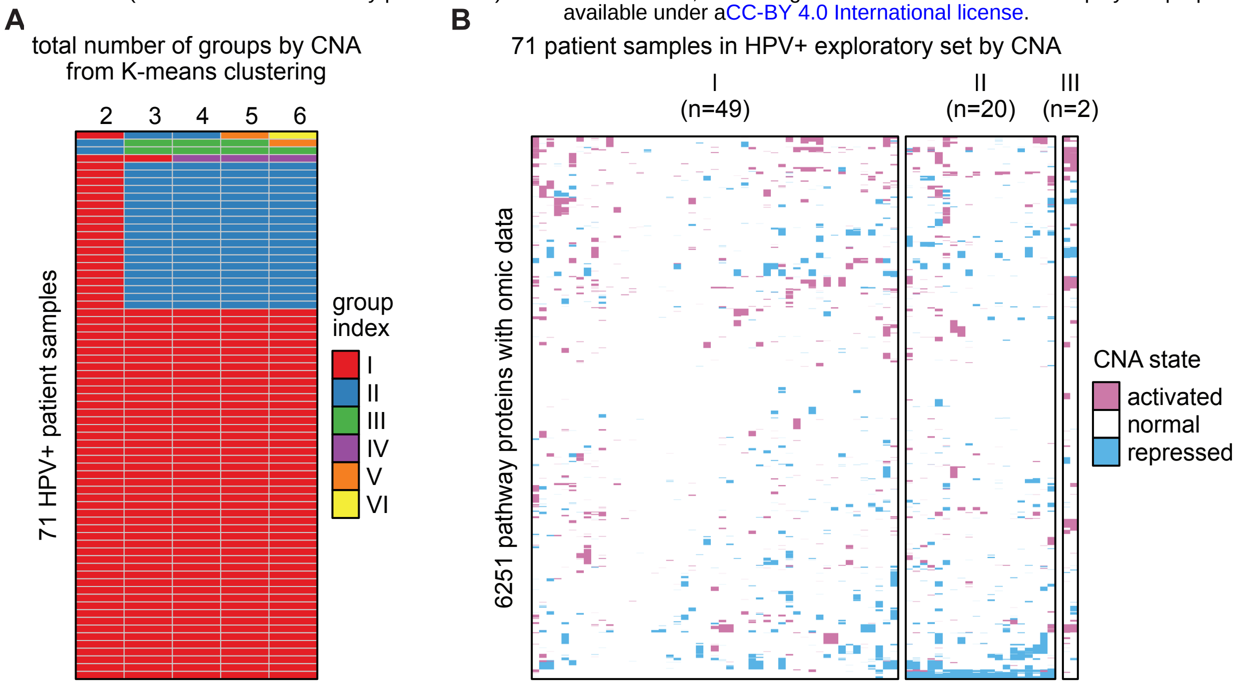

bioRxiv preprint doi: https://doi.org/10.1101/2024.06.15.599113; this version posted June 14, 2025. The copyright holder for this preprint (which was not certified by peer review) is the author/funder, who has granted bioRxiv a license to display the preprint in perpetuity. It is made available under aCC-BY 4.0 International license.

**Supplementary Figure 5**

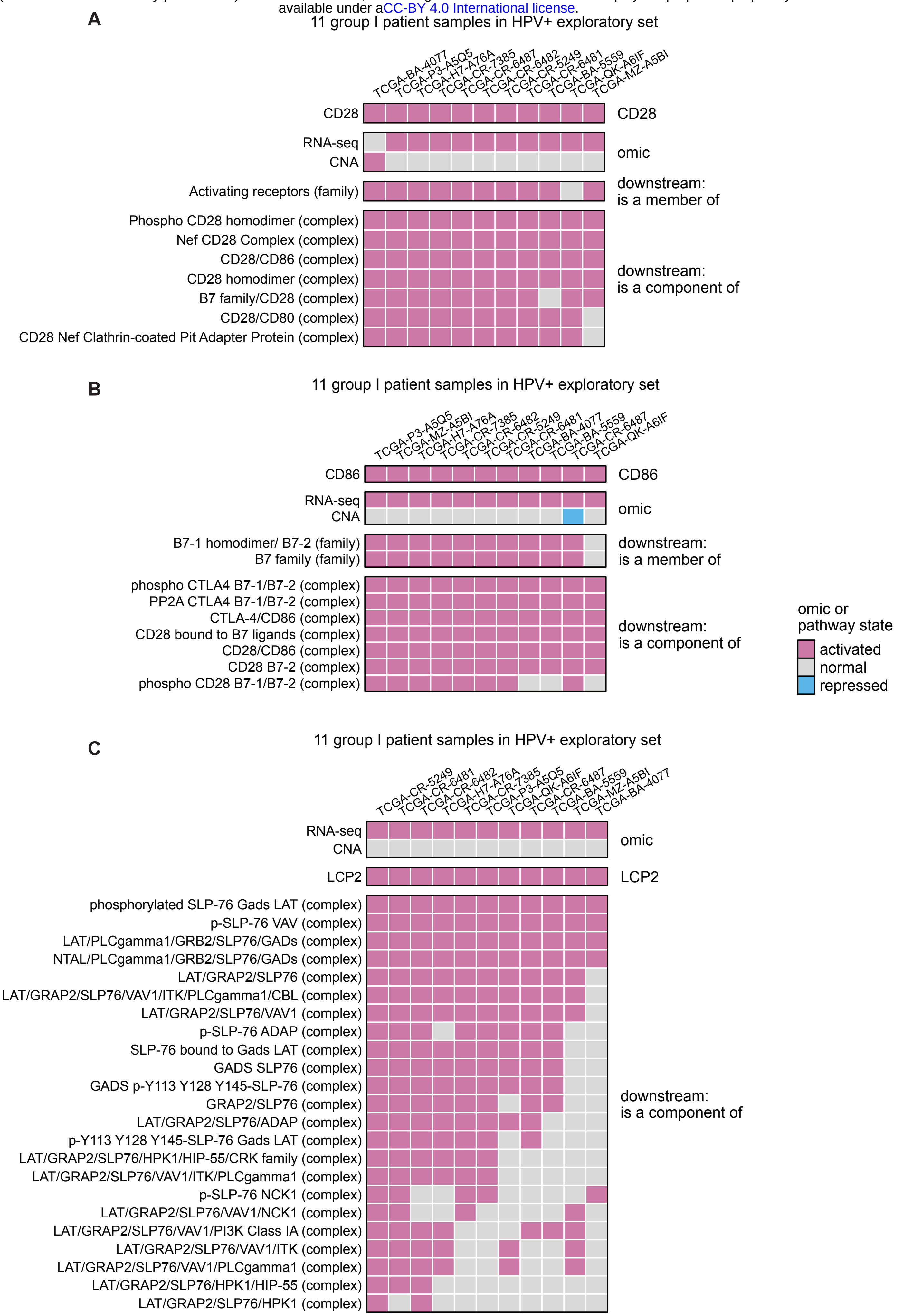

bioRxiv preprint doi: https://doi.org/10.1101/2024.06.15.599113; this version posted June 14, 2025. The copyright holder for this preprint (which was not certified by peer review) is the author/funder, who has granted bioRxiv a license to display the preprint in perpetuity. It is made available under aCC-BY 4.0 International license.

**Supplemental Figure 6**

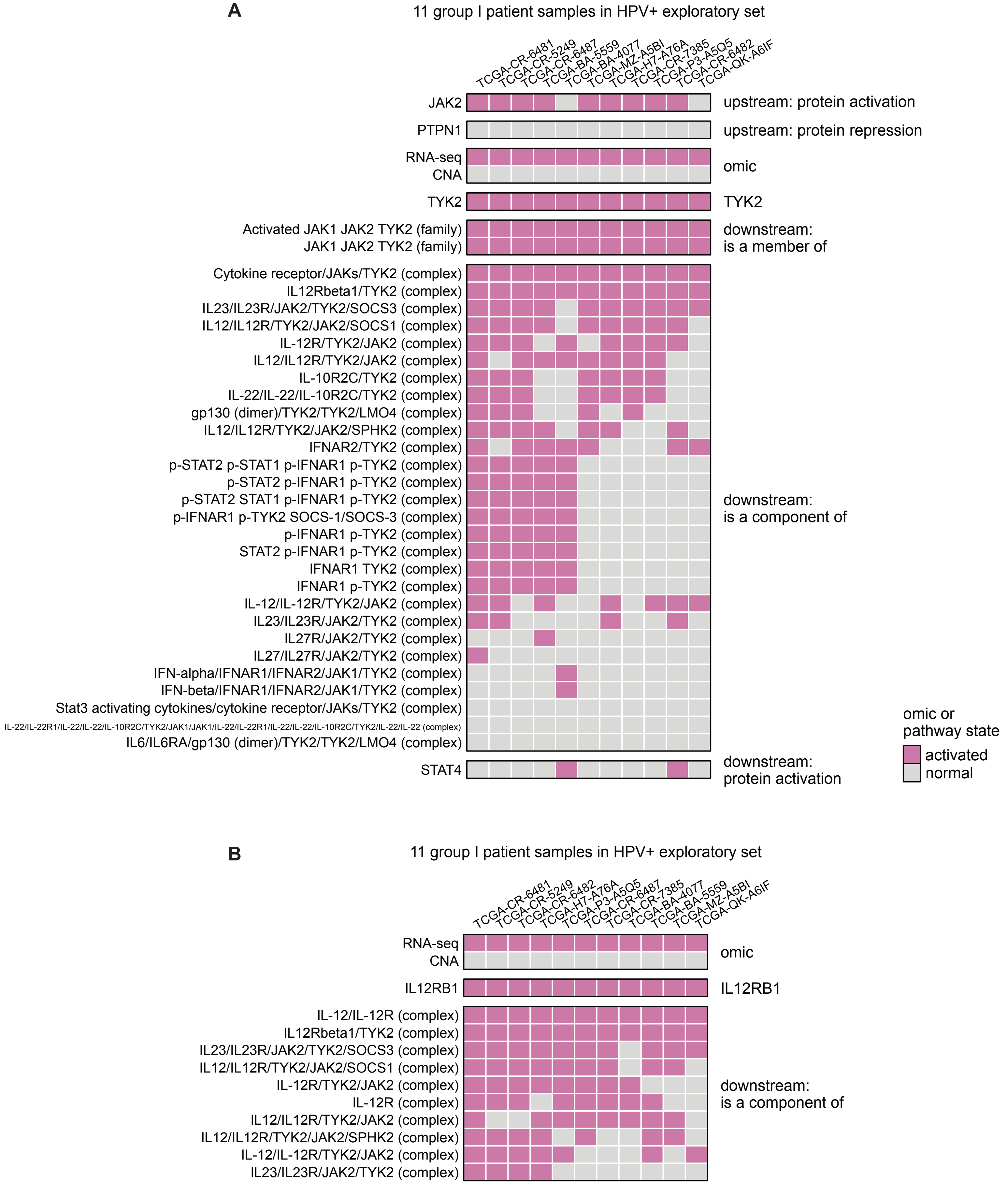

bioRxiv preprint doi: https://doi.org/10.1101/2024.06.15.599113; this version posted June 14, 2025. The copyright holder for this preprint (which was not certified by peer review) is the author/funder, who has granted bioRxiv a license to display the preprint in perpetuity. It is made available under aCC-BY 4.0 International license.

**Supplementary Figure 7**

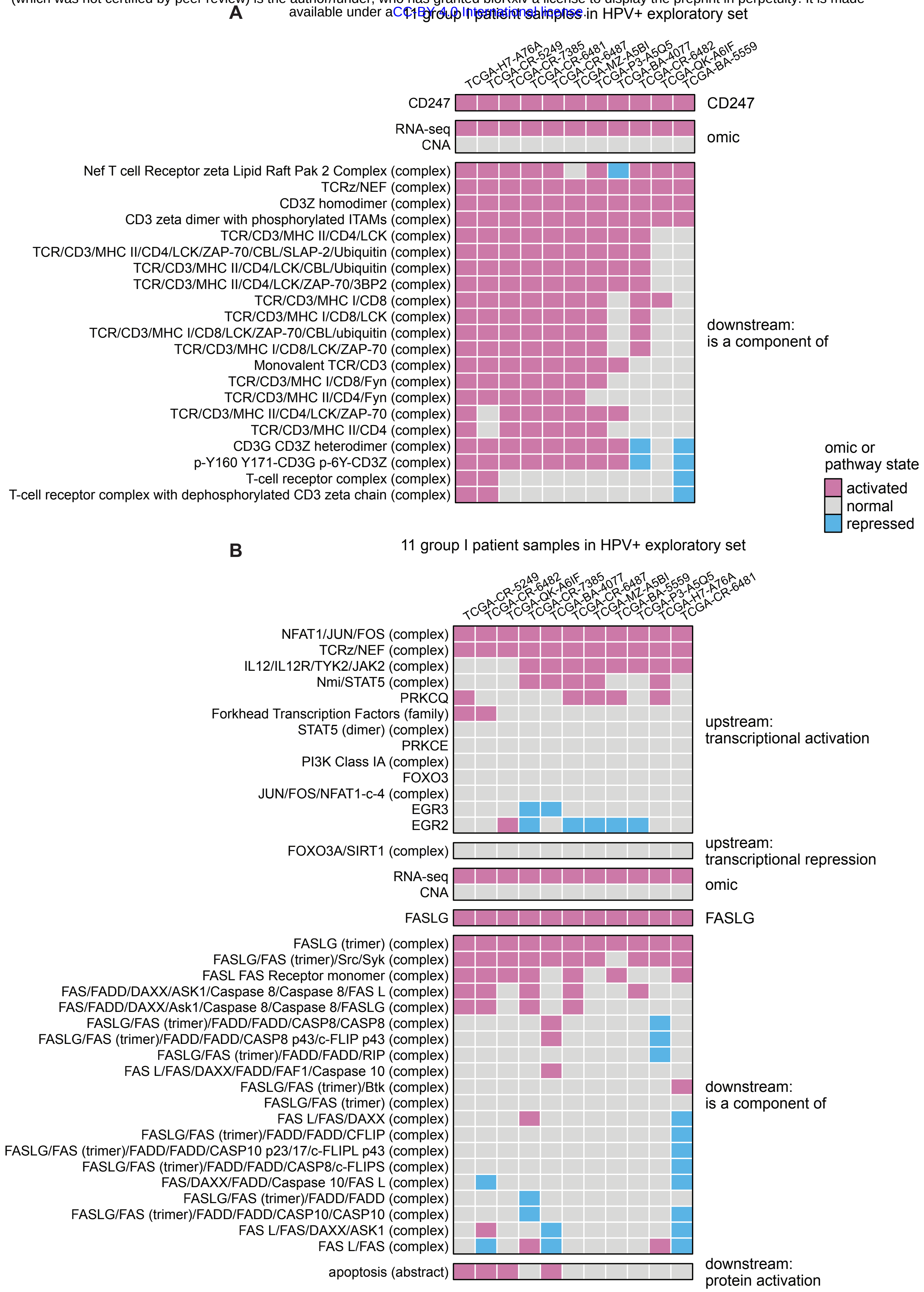

bioRxiv preprint doi: https://doi.org/10.1101/2024.06.15.599113; this version posted June 14, 2025. The copyright holder for this preprint (which was not certified by peer review) is the author/funder, who has granted bioRxiv a license to display the preprint in perpetuity. It is made available under aCC-BY 4.0 International license.

# Supplementary Figure 8

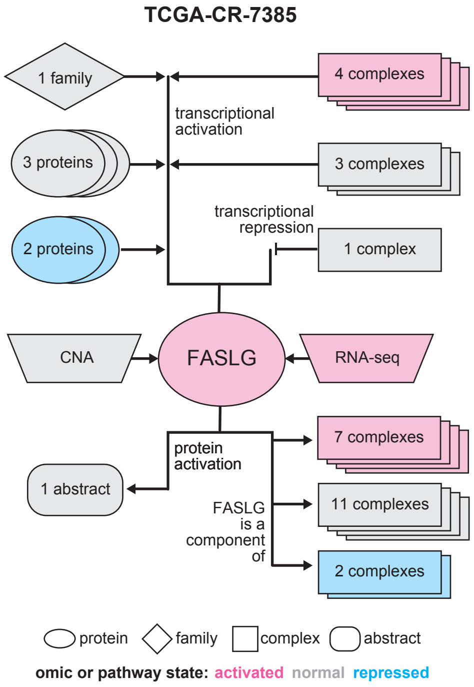

## Supplementary Figure 9

bioRxiv preprint doi: https://doi.org/10.1101/2024.06.15.599113; this version posted June 14, 2025. The copyright holder for this preprint (which was not certified by peer review) is the author/funder, who has granted bioRxiv a license to display the preprint in perpetuity. It is made available under aCC-BY 4.0 International license.

A B

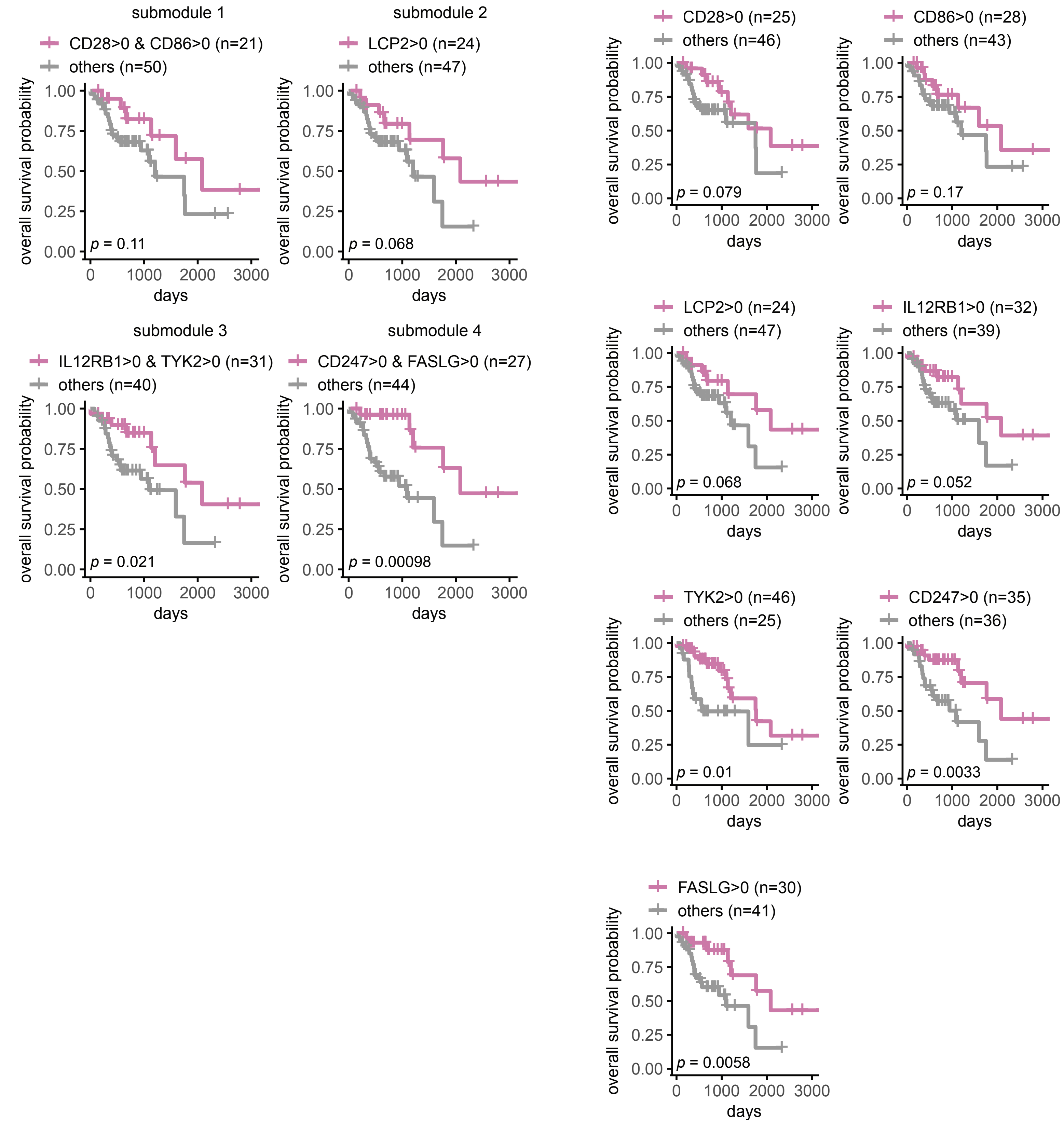

bioRxiv preprint doi: https://doi.org/10.1101/2024.06.15.599113; this version posted June 14, 2025. The copyright holder for this preprint (which was not certified by peer review) is the author/funder, who has granted bioRxiv a license to display the preprint in perpetuity. It is made available under aCC-BY 4.0 International license.

**Supplementary Figure 10**

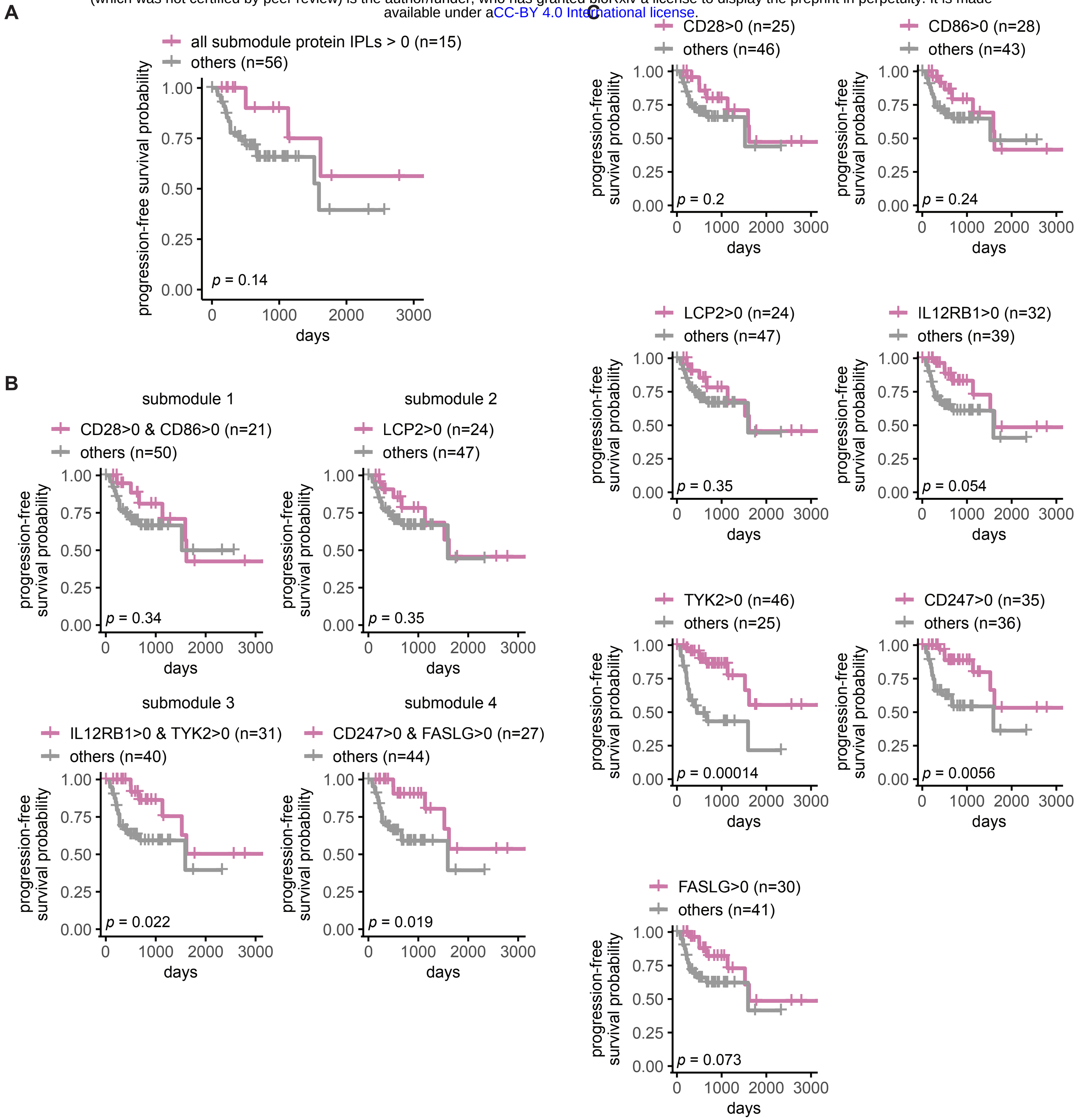

Supplementary Figure 11

bioRxiv preprint doi: https://doi.org/10.1101/2024.06.15.599113; this version posted June 14, 2025. The copyright holder for this preprint (which was not certified by peer review) is the author/funder, who has granted bioRxiv a license to display the preprint in perpetuity. It is made available under aCC-BY 4.0 International license.

A

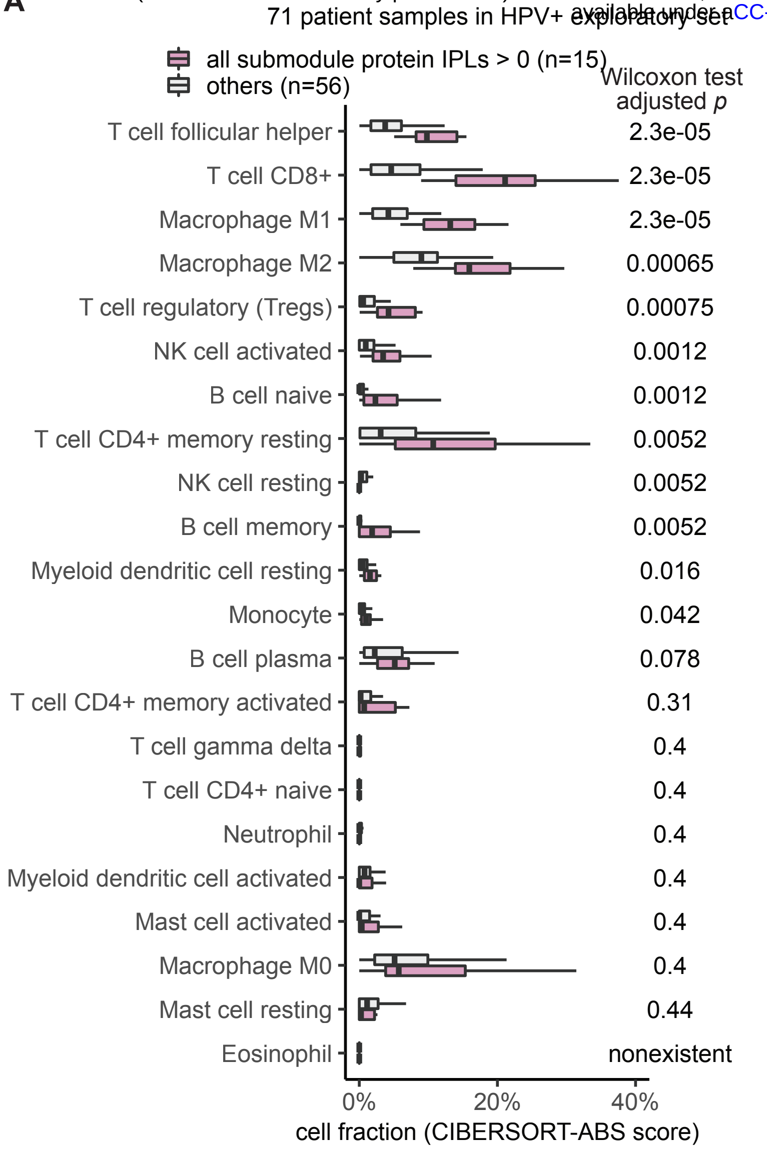


B

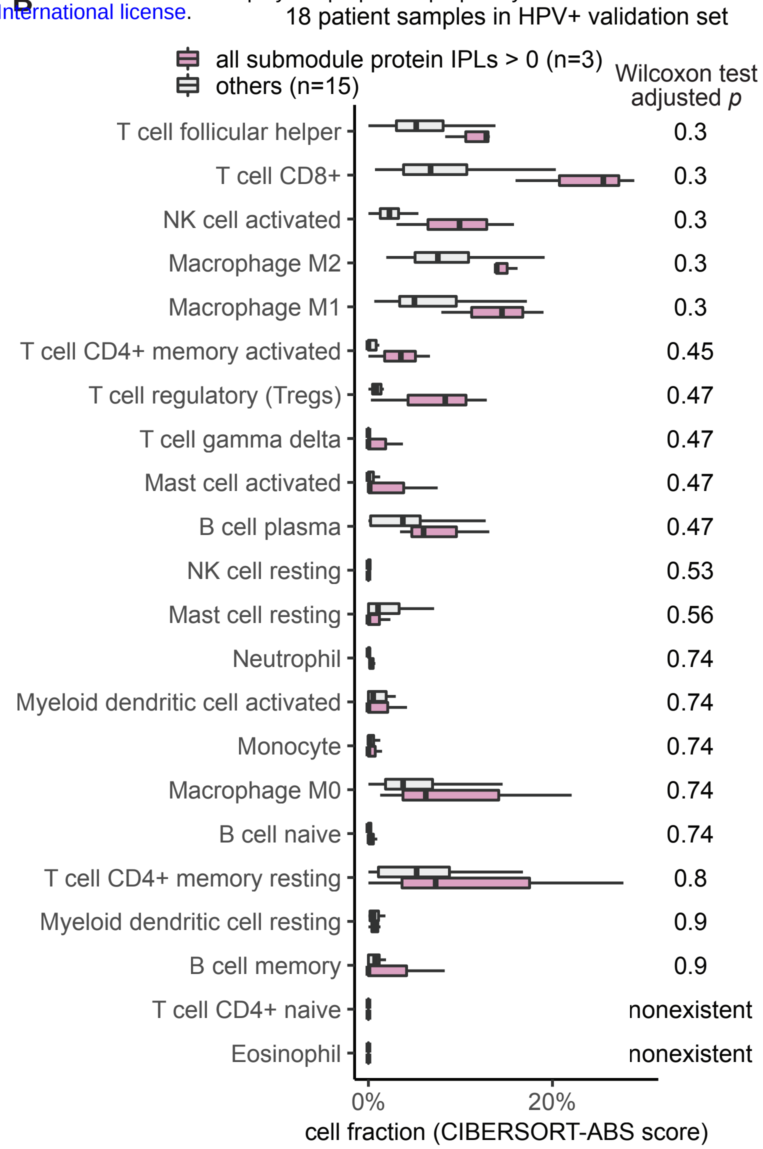

**Supplementary Figure 12**

bioRxiv preprint doi: https://doi.org/10.1101/2024.06.15.599113; this version posted June 14, 2025. The copyright holder for this preprint (which was not certified by peer review) is the author/funder, who has granted bioRxiv a license to display the preprint in perpetuity. It is made available under aCC-BY 4.0 International license.

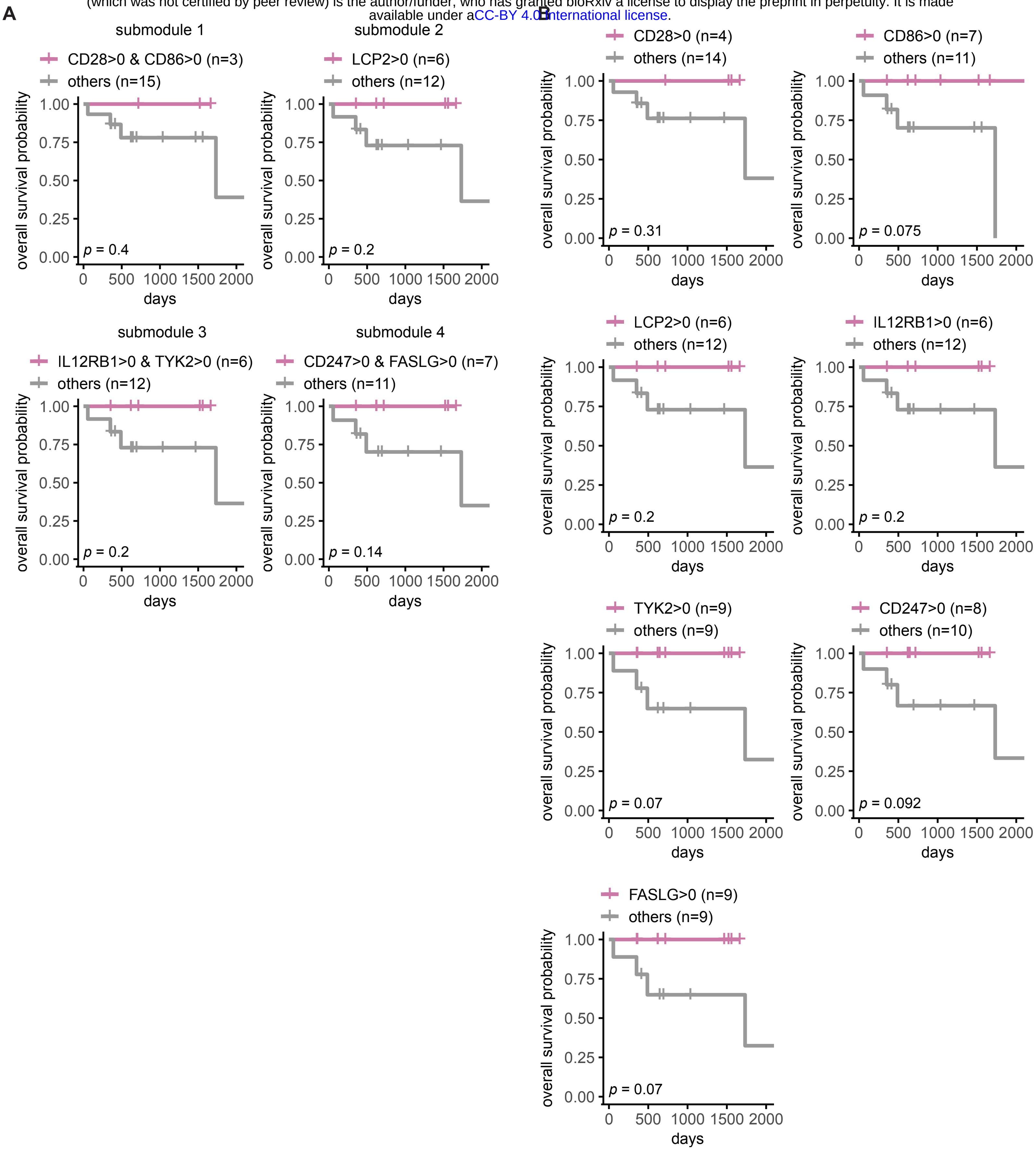

**Supplementary Figure 13**

bioRxiv preprint doi: https://doi.org/10.1101/2024.06.15.599113; this version posted June 14, 2025. The copyright holder for this preprint (which was not certified by peer review) is the author/funder, who has granted bioRxiv a license to display the preprint in perpetuity. It is made available under aCC-BY 4.0 International license.

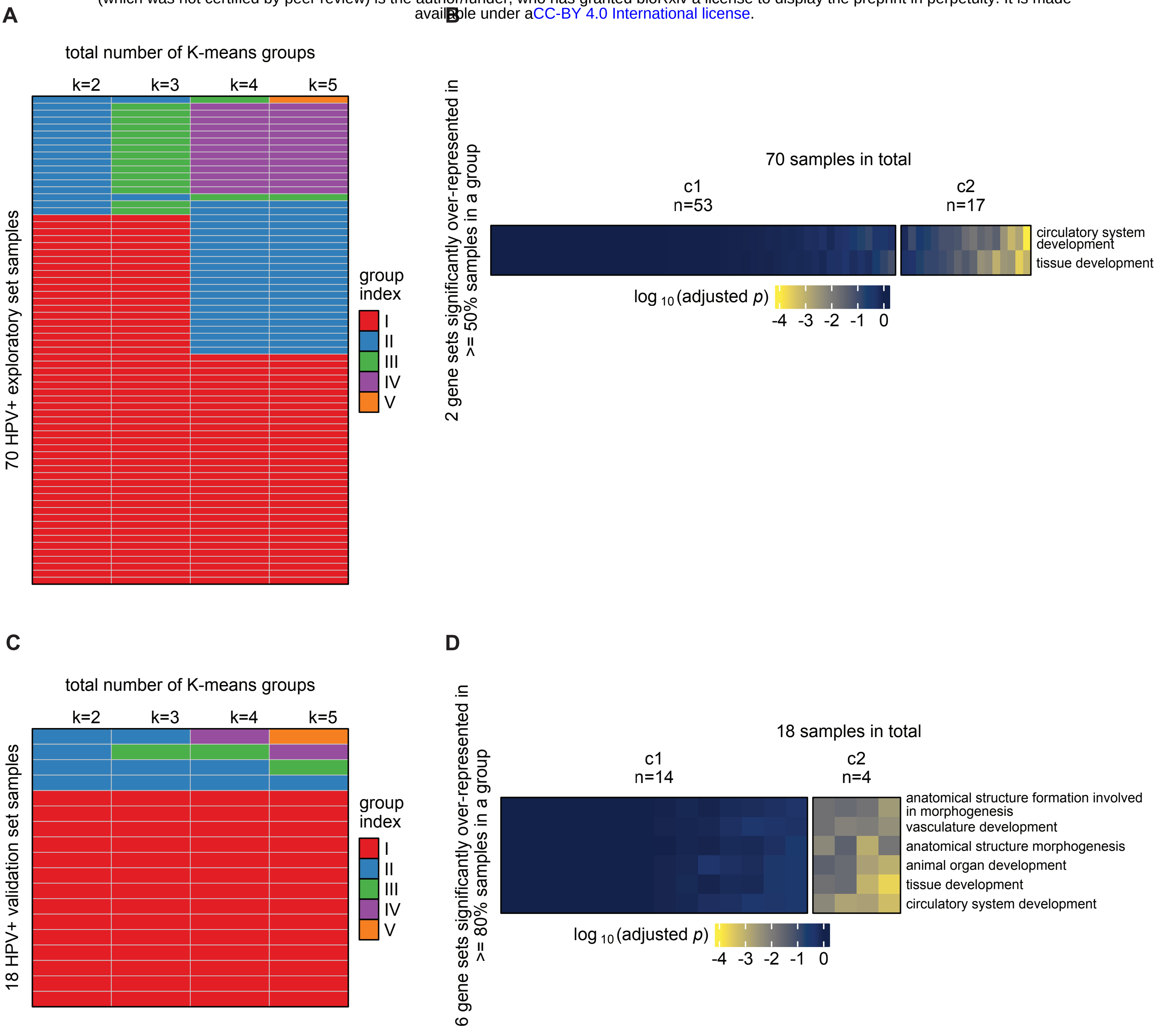

## Supplementary Figure 14

bioRxiv preprint doi: https://doi.org/10.1101/2024.06.15.599113; this version posted June 14, 2025. The copyright holder for this preprint (which was not certified by peer review) is the author/funder, who has granted bioRxiv a license to display the preprint in perpetuity. It is made available under aCC-BY 4.0 International license.

**A**

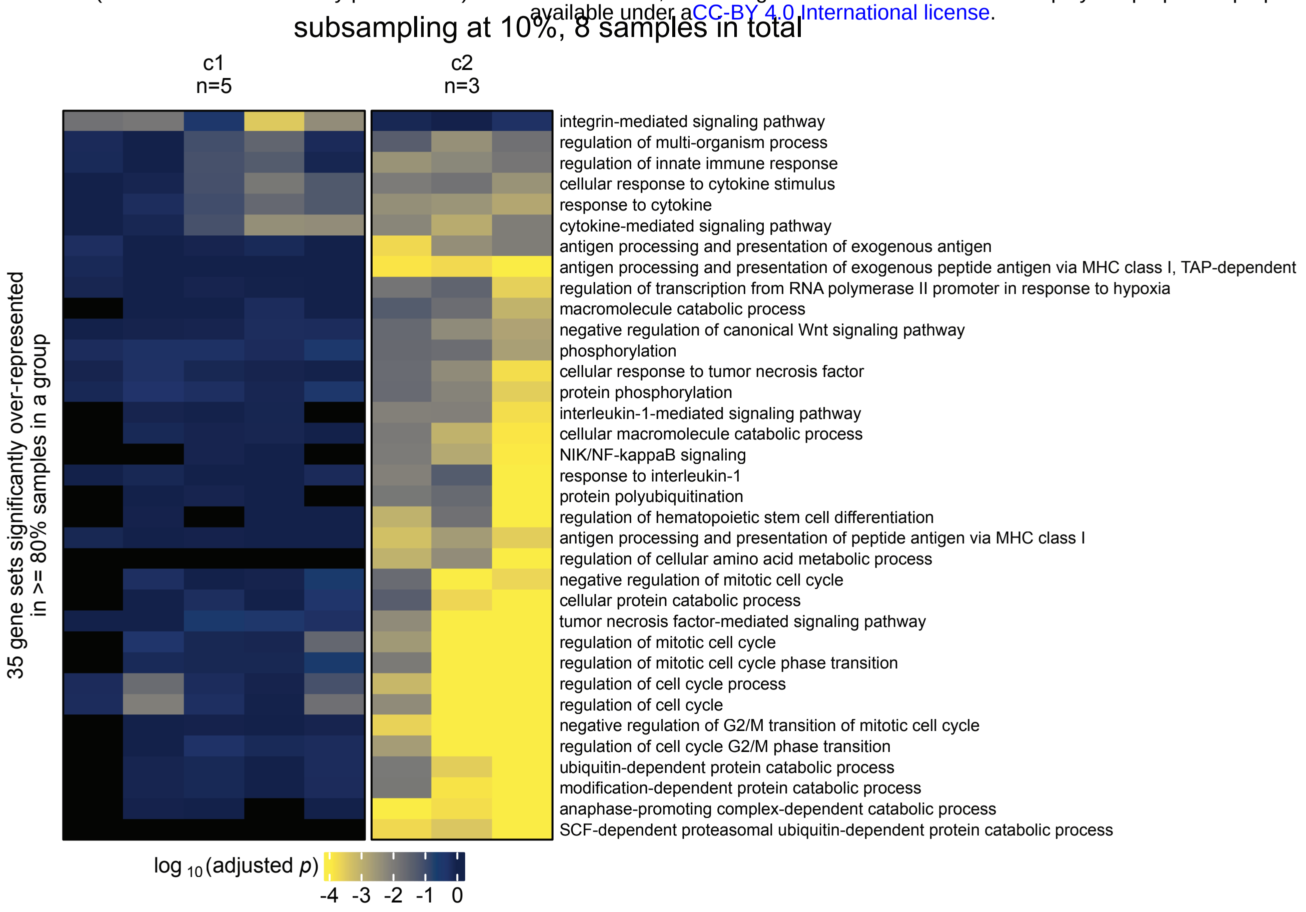


**B**

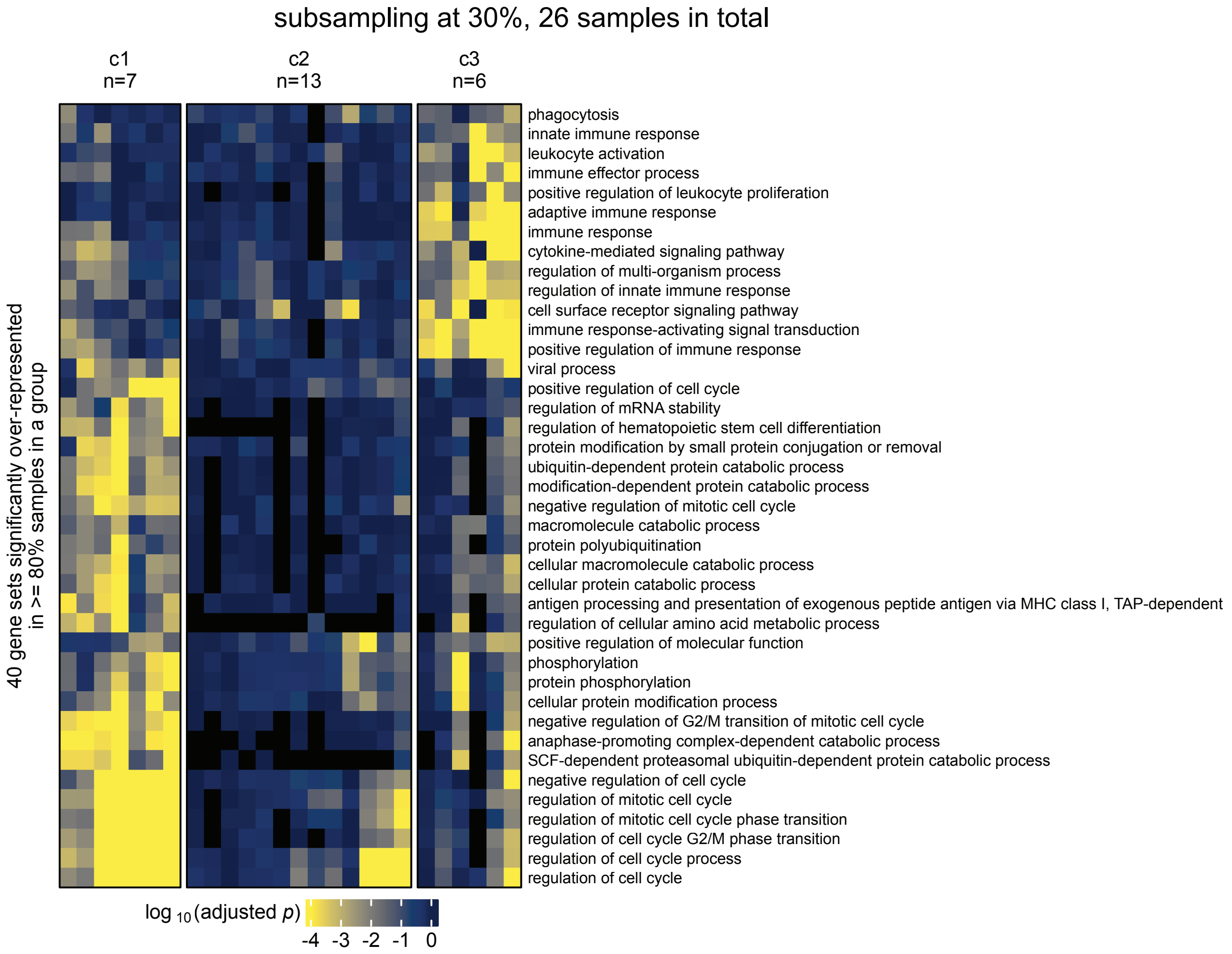


continued on the next page for panel C

bioRxiv preprint doi: https://doi.org/10.1101/2024.06.15.599113; this version posted June 14, 2025. The copyright holder for this preprint (which was not certified by peer review) is the author/funder, who has granted bioRxiv a license to display the preprint in perpetuity. It is made available under aCC-BY 4.0 International license.

**Supplementary Figure 14**

**C**

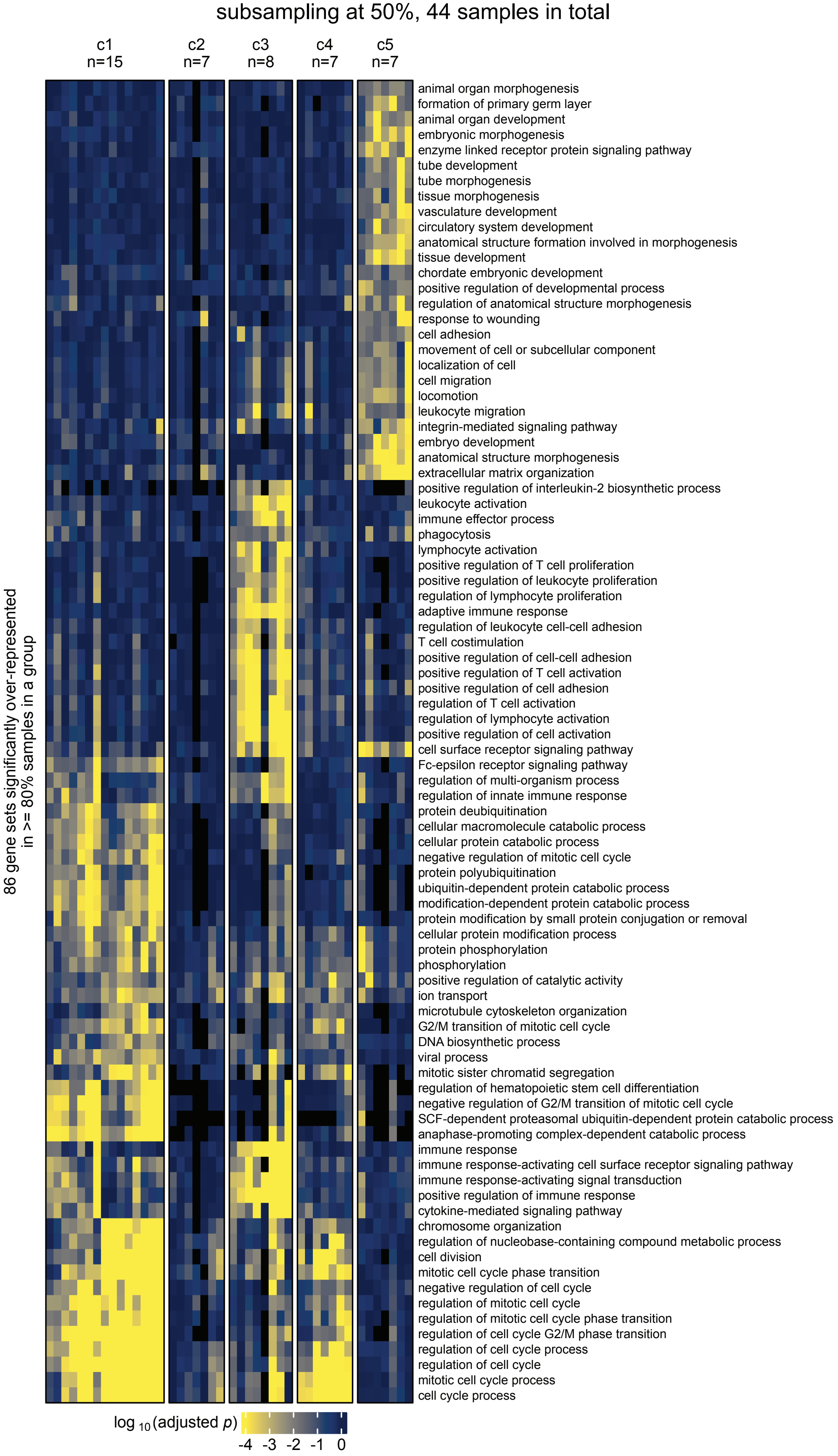

# Supplementary Figure 15

bioRxiv preprint doi: https://doi.org/10.1101/2024.06.15.599113; this version posted June 14, 2025. The copyright holder for this preprint (which was not certified by peer review) is the author/funder, who has granted bioRxiv a license to display the preprint in perpetuity. It is made available under aCC-BY 4.0 International license.

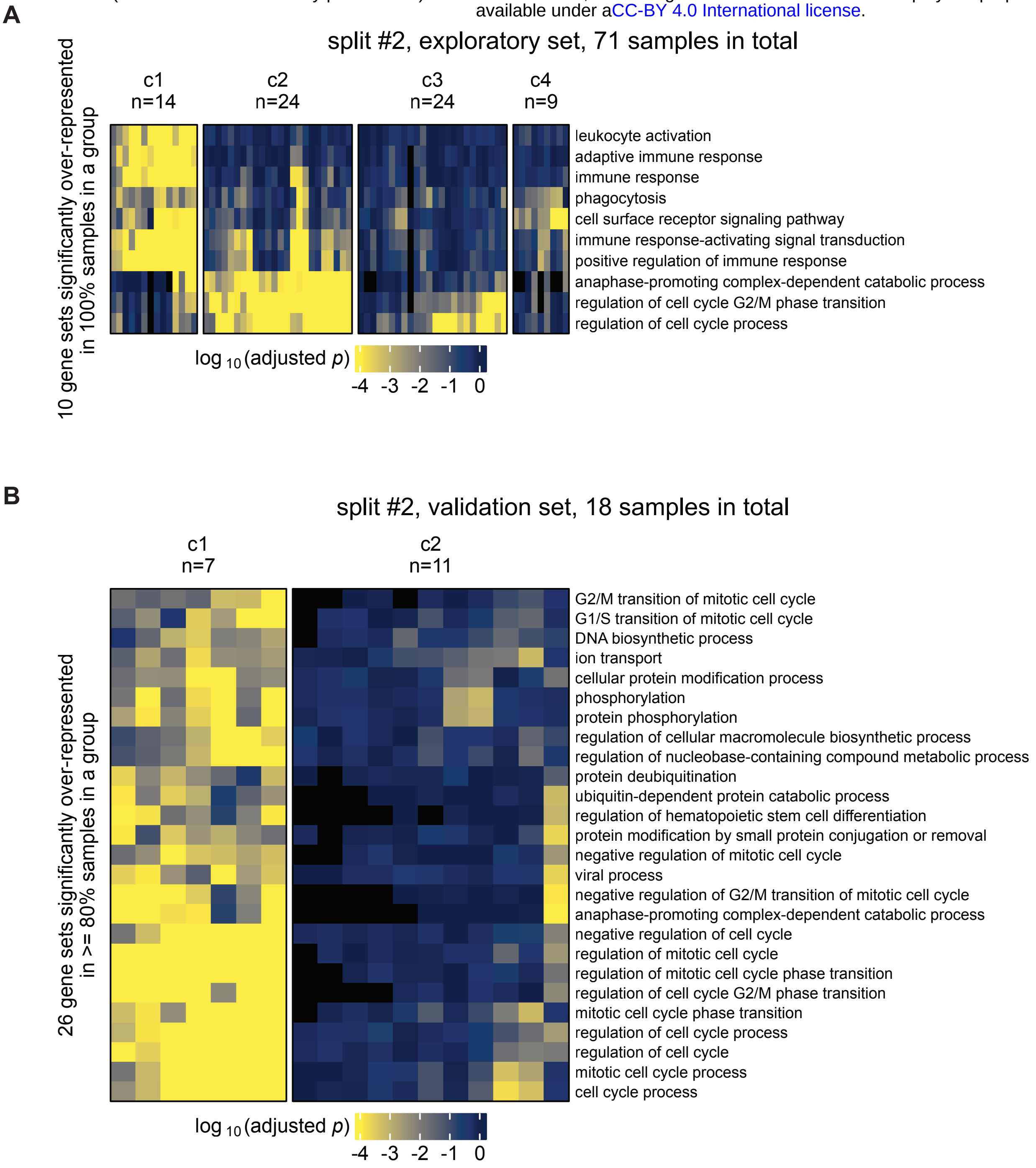


continued on the next page for other panels

Supplementary Figure 15

bioRxiv preprint doi: https://doi.org/10.1101/2024.06.15.599113; this version posted June 14, 2025. The copyright holder for this preprint (which was not certified by peer review) is the author/funder, who has granted bioRxiv a license to display the preprint in perpetuity. It is made available under aCC-BY 4.0 International license.

C

split #3, exploratory set, 71 samples in total

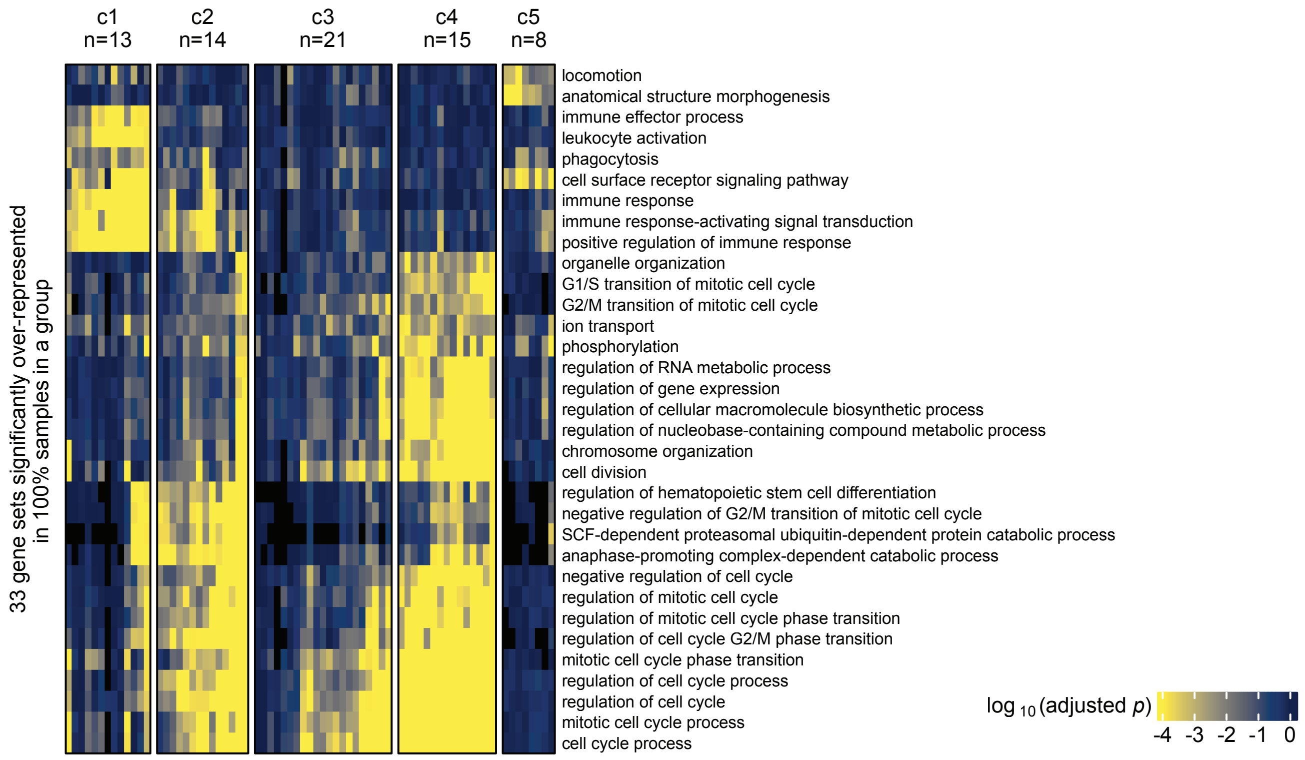


D

split #3, validation set,18 samples in total

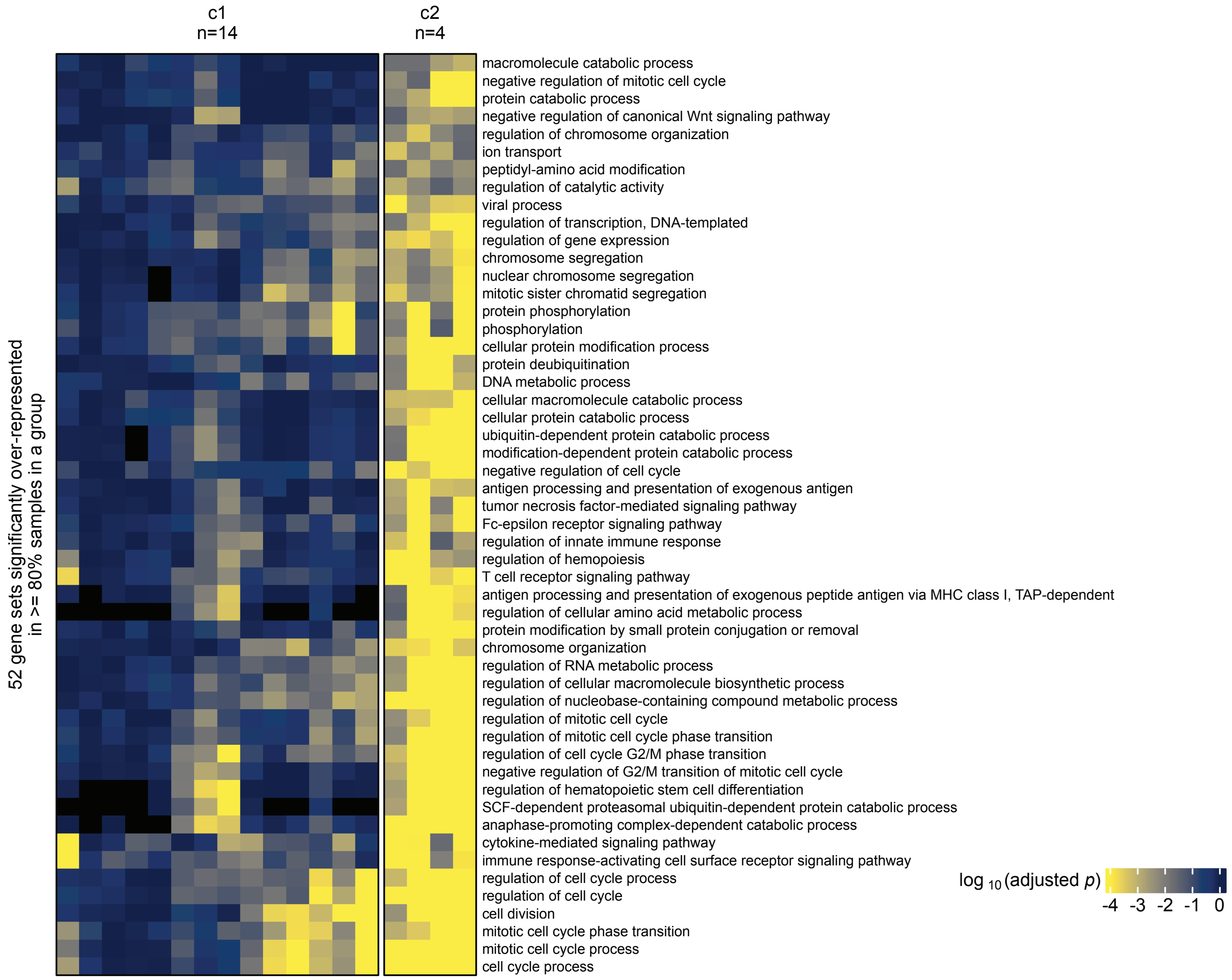


continued on the next page for other panels

bioRxiv preprint doi: https://doi.org/10.1101/2024.06.15.599113; this version posted June 14, 2025. The copyright holder for this preprint (which was not certified by peer review) is the author/funder, who has granted bioRxiv a license to display the preprint in perpetuity. It is made available under aCC-BY 4.0 International license.

**Supplementary Figure 15**

**E**

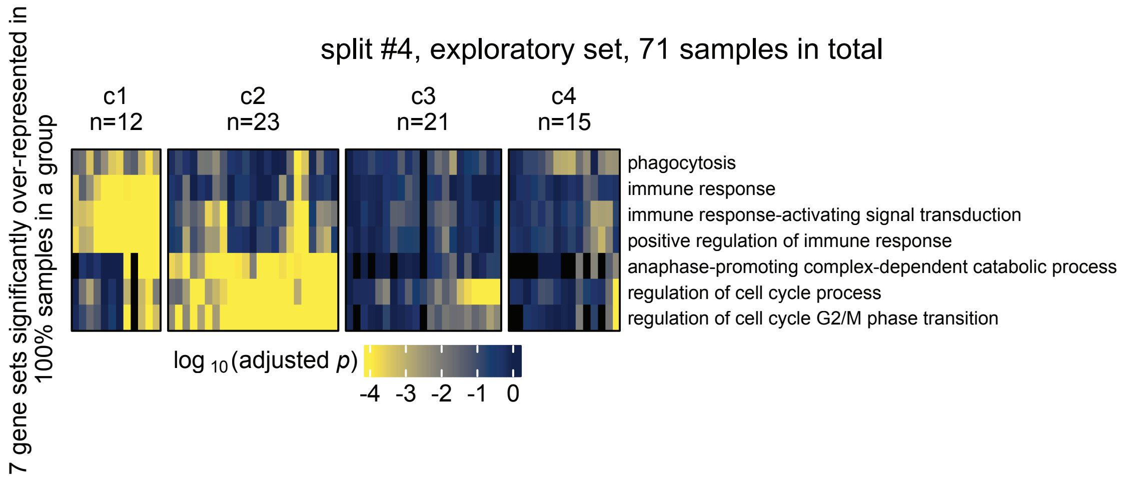


**F**

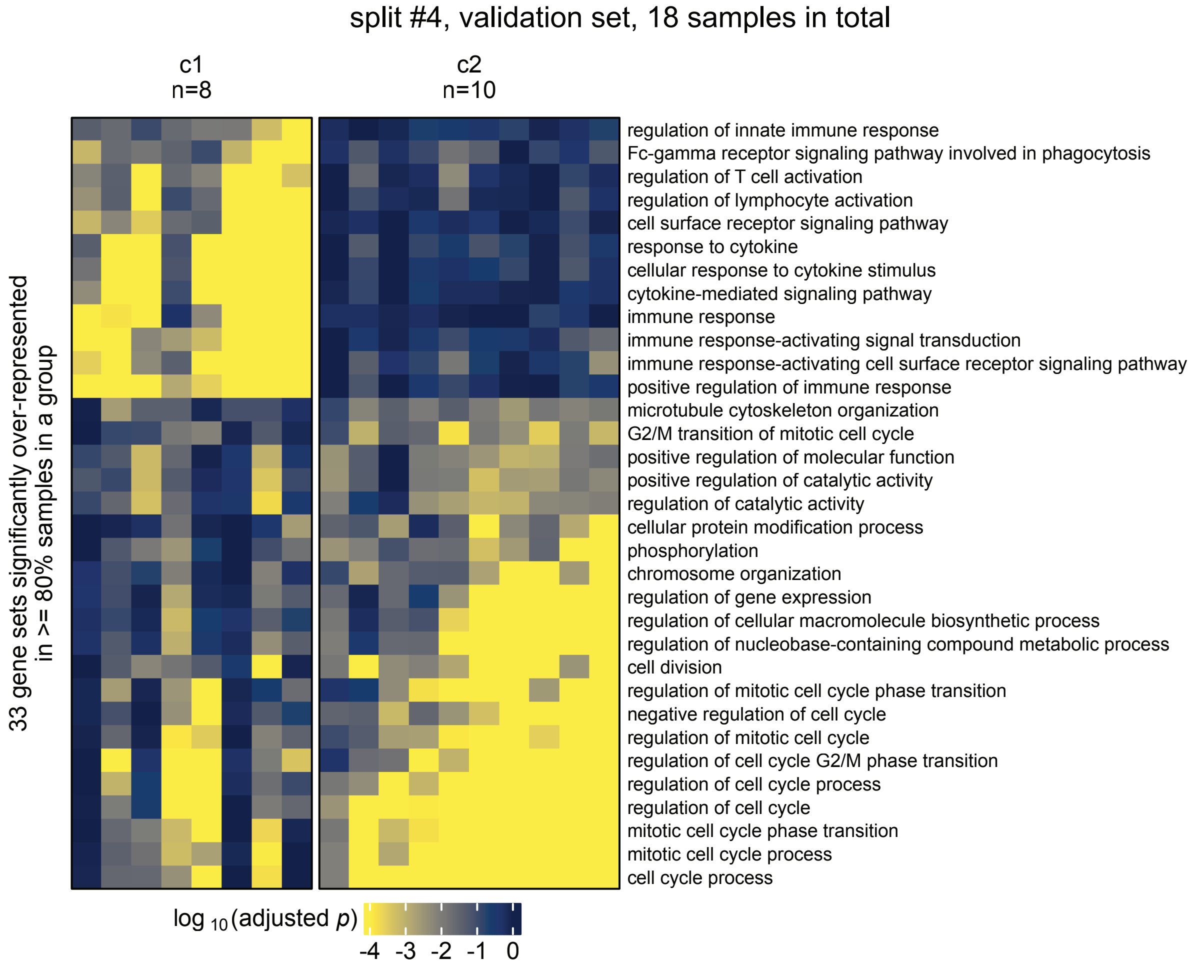


continued on the next page for other panels

Supplementary Figure 15

bioRxiv preprint doi: https://doi.org/10.1101/2024.06.15.599113; this version posted June 14, 2025. The copyright holder for this preprint (which was not certified by peer review) is the author/funder, who has granted bioRxiv a license to display the preprint in perpetuity. It is made available under aCC-BY 4.0 International license.

G

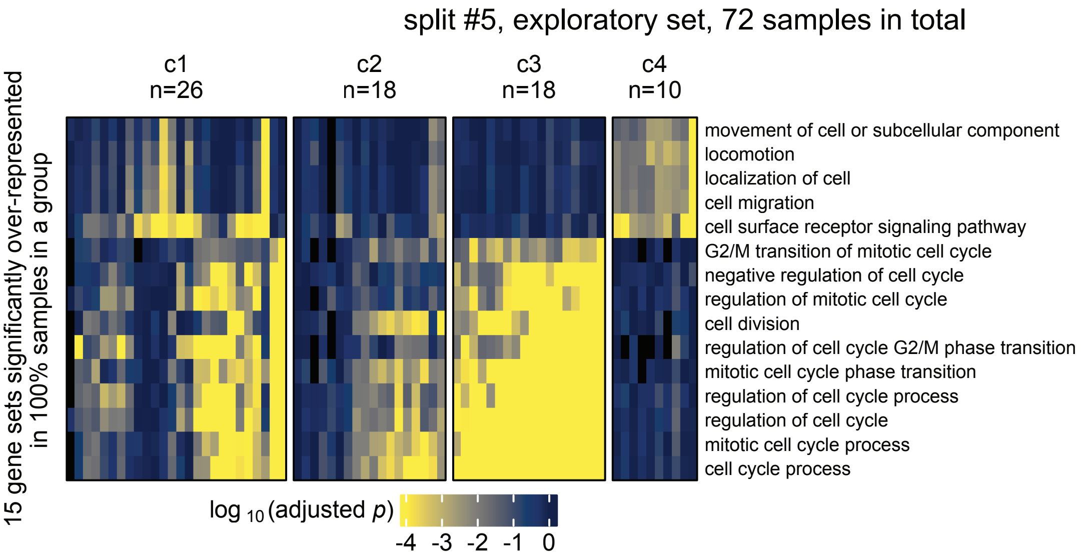


H

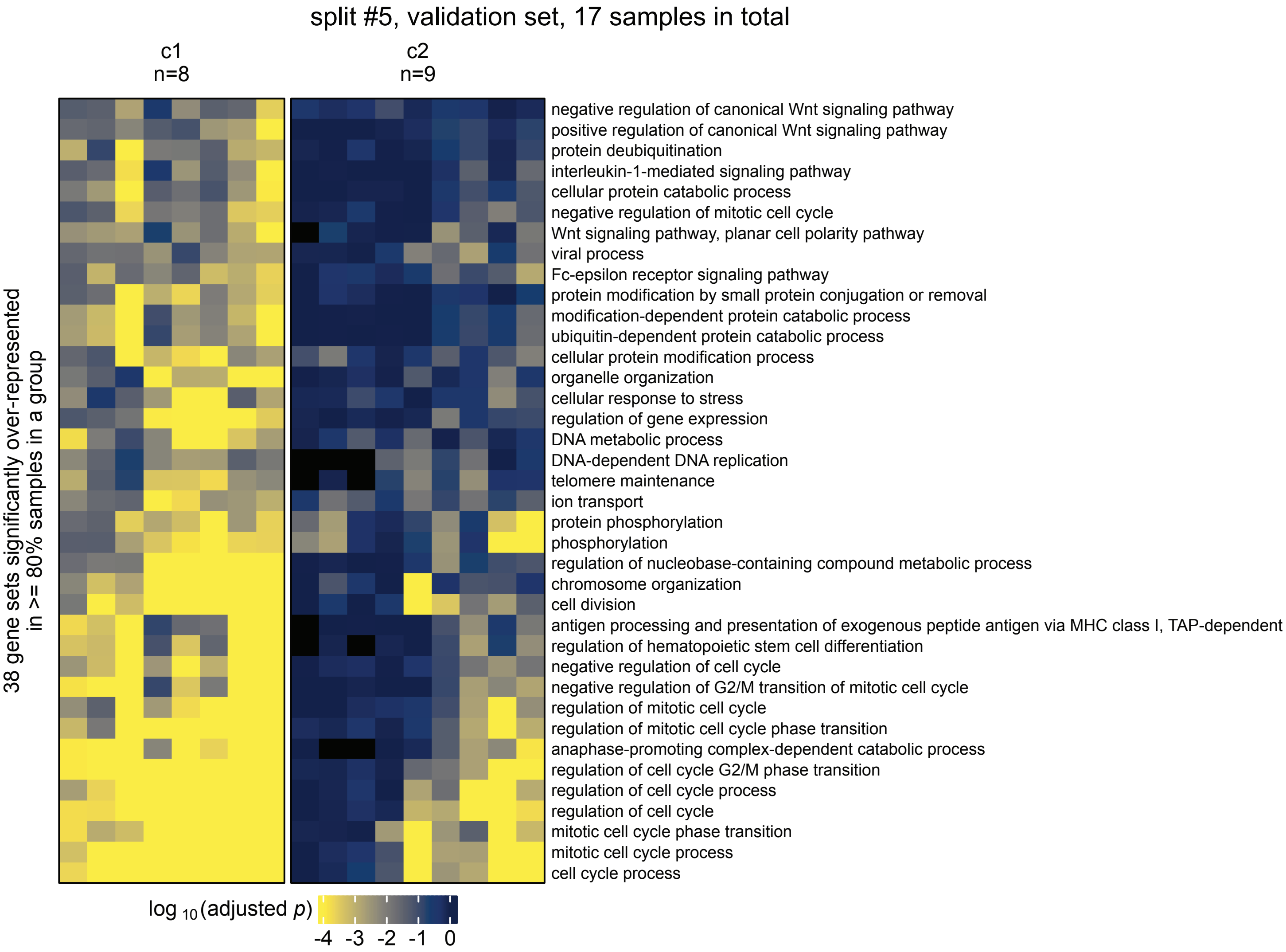

**Supplementary Figure 16**

bioRxiv preprint doi: https://doi.org/10.1101/2024.06.15.599113; this version posted June 14, 2025. The copyright holder for this preprint (which was not certified by peer review) is the author/funder, who has granted bioRxiv a license to display the preprint in perpetuity. It is made available under aCC-BY 4.0 International license.

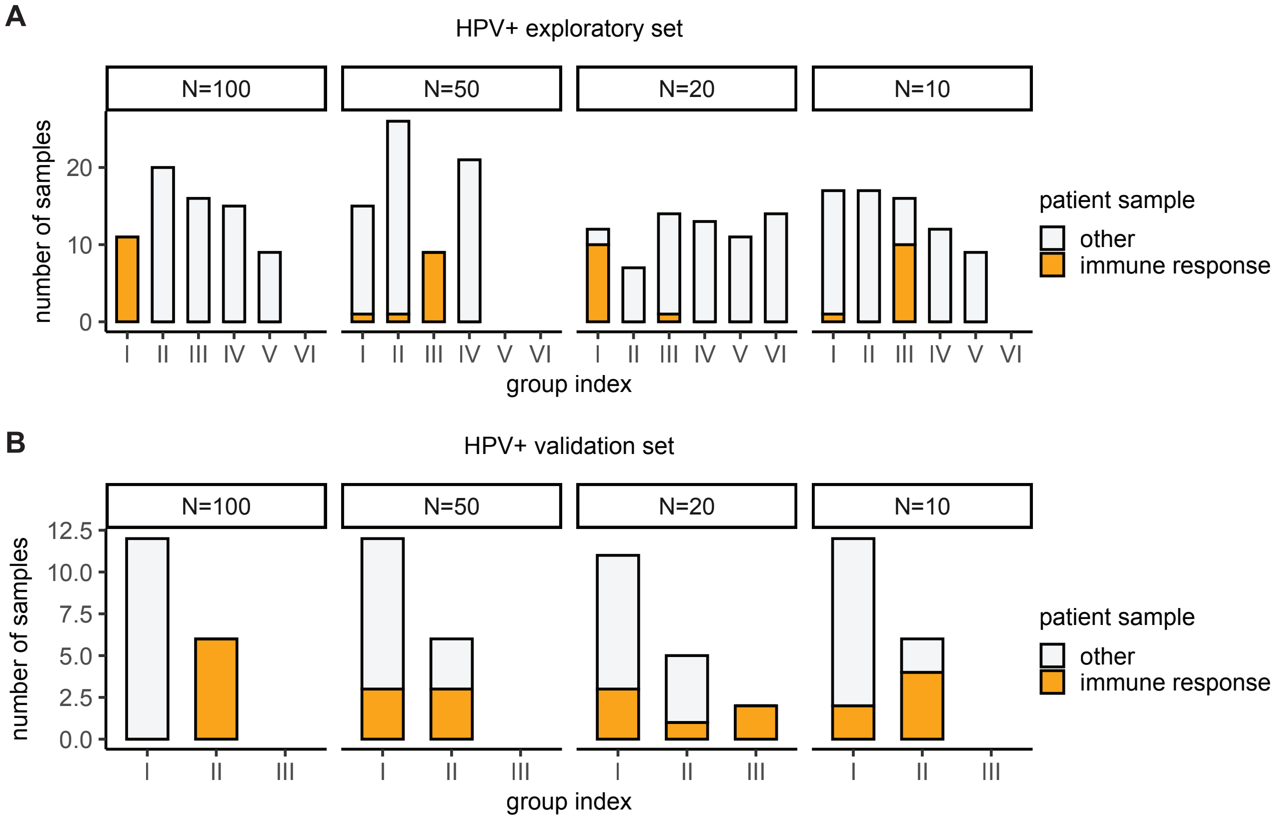

**Supplementary Figure 17**

bioRxiv preprint doi: https://doi.org/10.1101/2024.06.15.599113; this version posted June 14, 2025. The copyright holder for this preprint (which was not certified by peer review) is the author/funder, who has granted bioRxiv a license to display the preprint in perpetuity. It is made available under aCC-BY 4.0 International license.

**A**

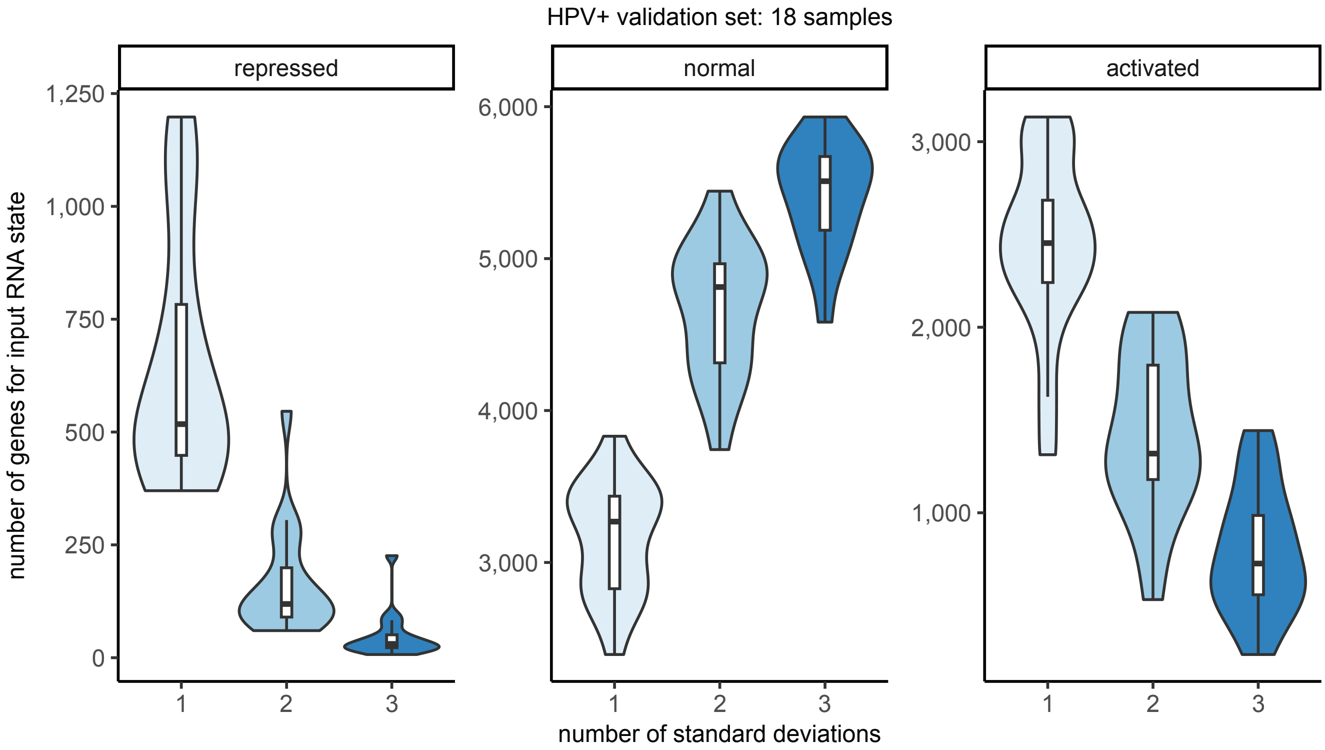


**B**

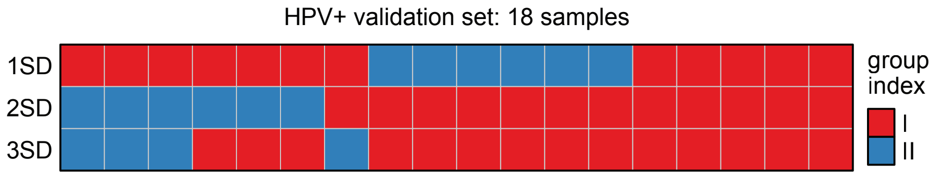


continued on the next page for panel C & D

bioRxiv preprint doi: https://doi.org/10.1101/2024.06.15.599113; this version posted June 14, 2025. The copyright holder for this preprint (which was not certified by peer review) is the author/funder, who has granted bioRxiv a license to display the preprint in perpetuity. It is made available under aCC-BY 4.0 International license.

**Supplementary Figure 17**

**C**

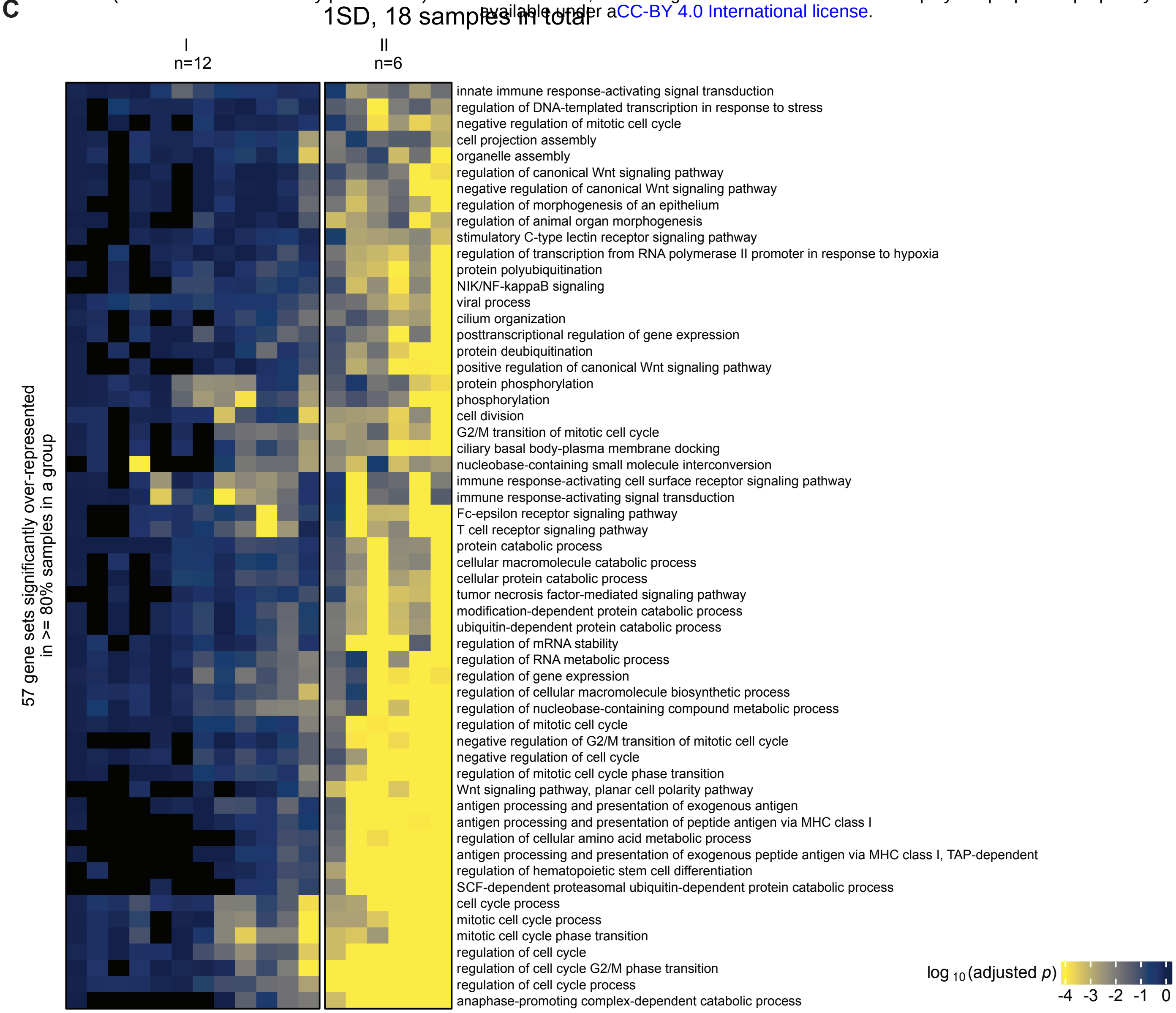


**D**

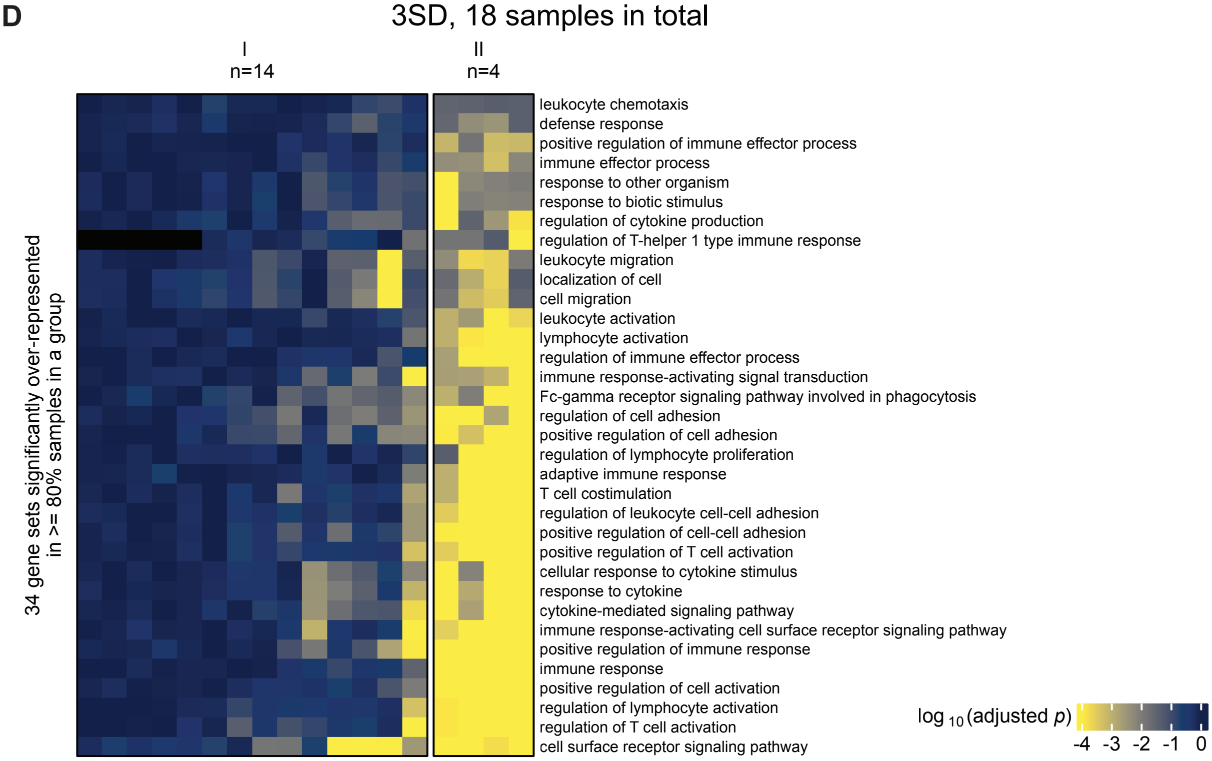

bioRxiv preprint doi: https://doi.org/10.1101/2024.06.15.599113; this version posted June 14, 2025. The copyright holder for this preprint (which was not certified by peer review) is the author/funder, who has granted bioRxiv a license to display the preprint in perpetuity. It is made available under aCC-BY 4.0 International license.

**Supplementary Figure 18**

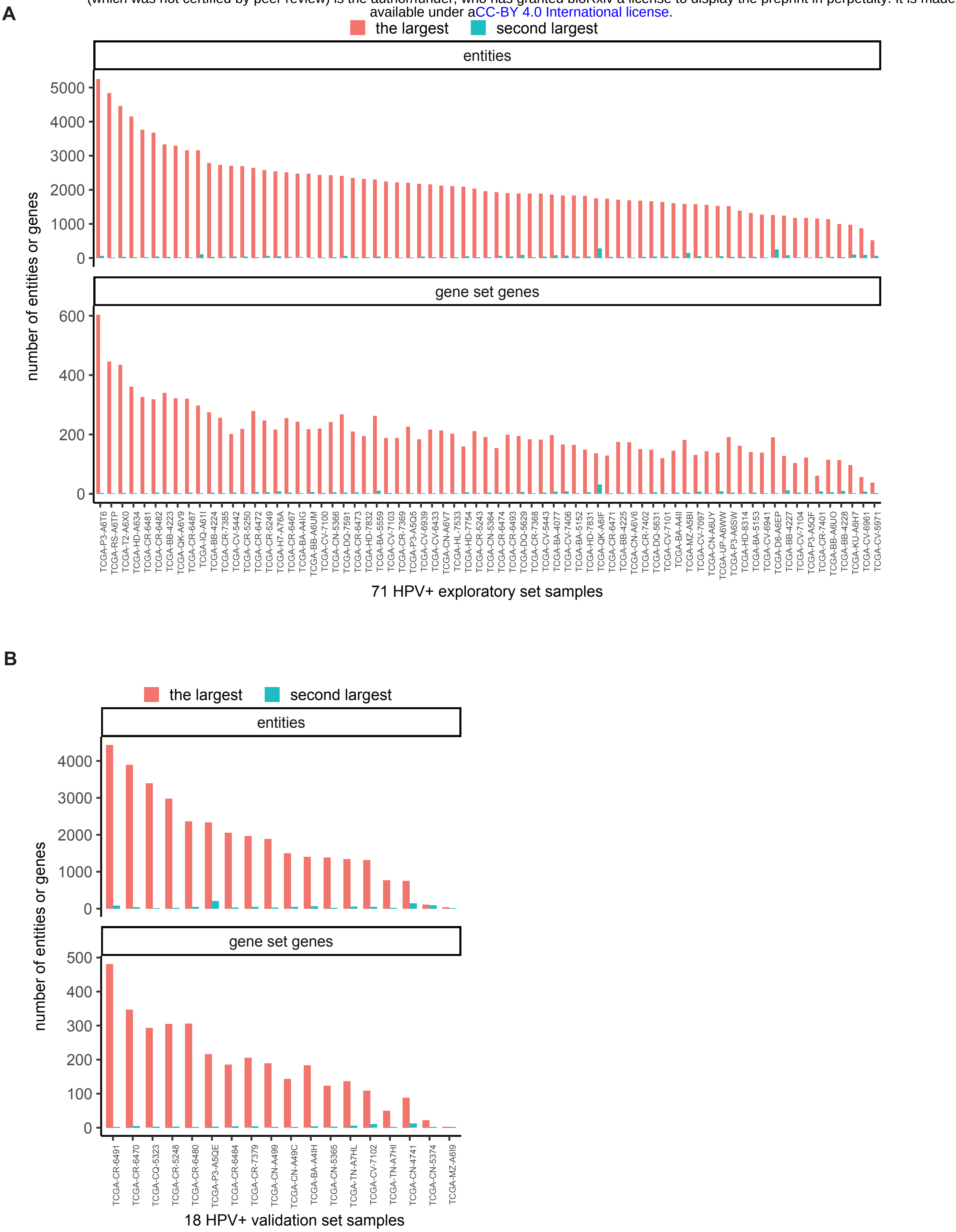

## Supplementary Figure 19

bioRxiv preprint doi: https://doi.org/10.1101/2024.06.15.599113; this version posted June 14, 2025. The copyright holder for this preprint (which was not certified by peer review) is the author/funder, who has granted bioRxiv a license to display the preprint in perpetuity. It is made available under aCC-BY 4.0 International license.

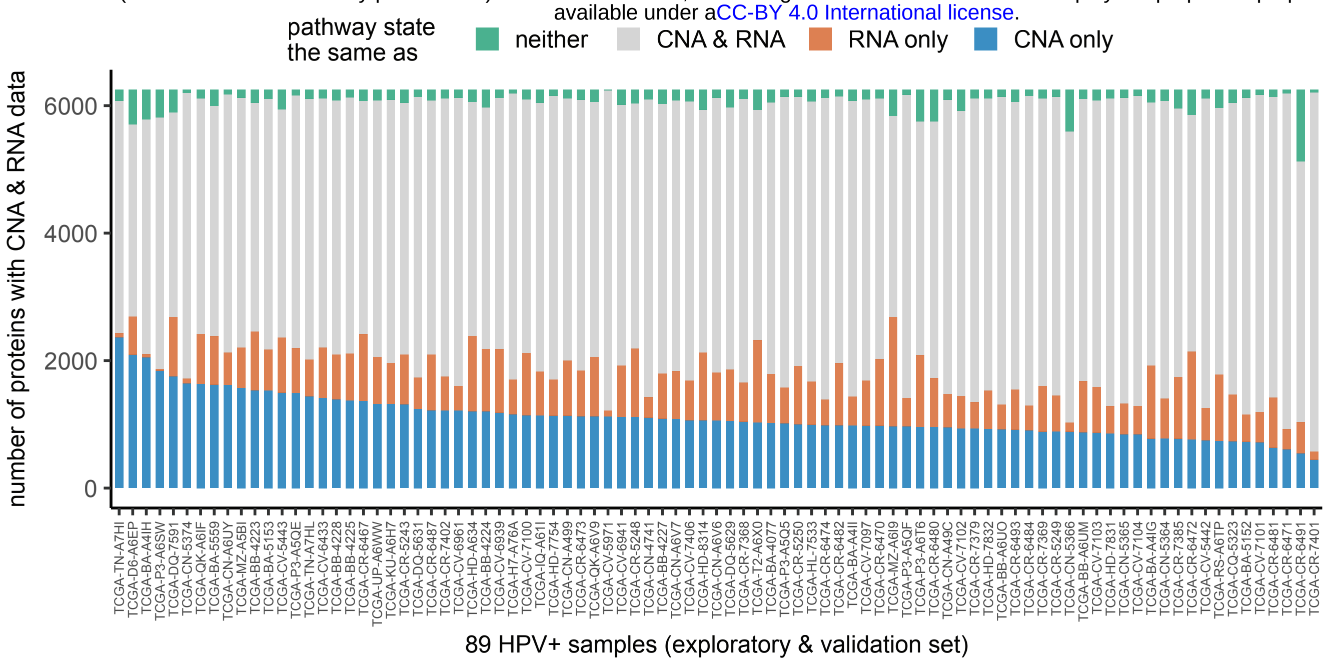

bioRxiv preprint doi: https://doi.org/10.1101/2024.06.15.599113; this version posted June 14, 2025. The copyright holder for this preprint (which was not certified by peer review) is the author/funder, who has granted bioRxiv a license to display the preprint in perpetuity. It is made available under aCC-BY 4.0 International license.

**Supplementary Figure 20**

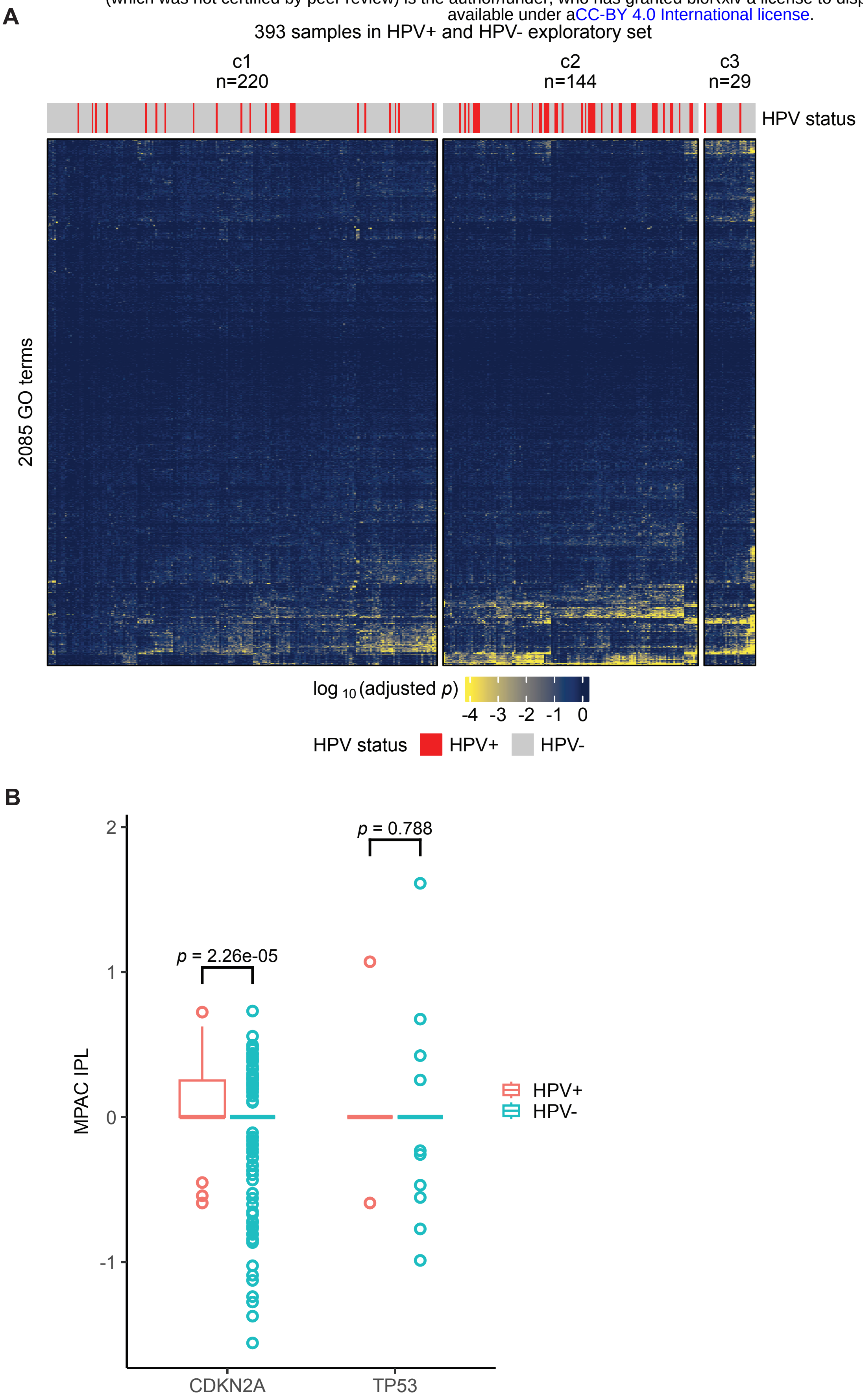

bioRxiv preprint doi: https://doi.org/10.1101/2024.06.15.599113; this version posted June 14, 2025. The copyright holder for this preprint (which was not certified by peer review) is the author/funder, who has granted bioRxiv a license to display the preprint in perpetuity. It is made available under aCC-BY 4.0 International license.

## Supplementary Figure 21

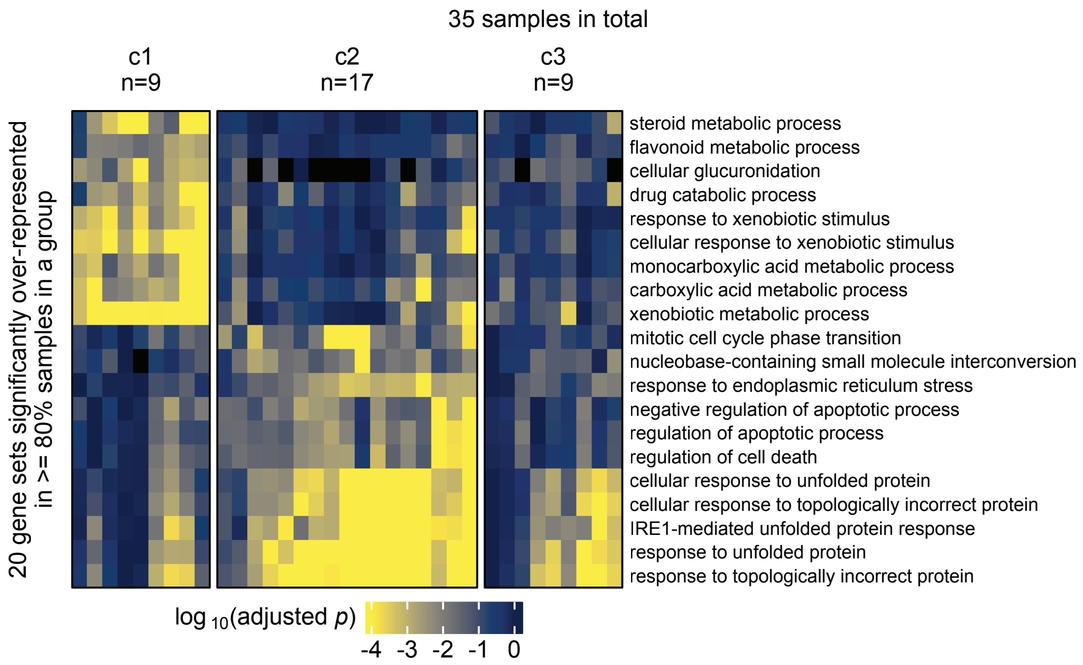

## Supplementary Figure 22

bioRxiv preprint doi: https://doi.org/10.1101/2024.06.15.599113; this version posted June 14, 2025. The copyright holder for this preprint (which was not certified by peer review) is the author/funder, who has granted bioRxiv a license to display the preprint in perpetuity. It is made available under aCC-BY 4.0 International license.

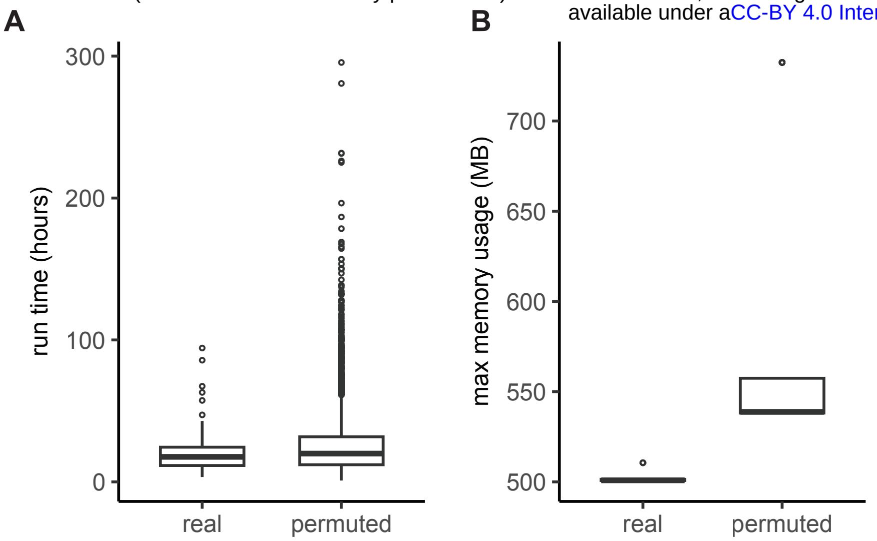

Supplement: 1 [file NIHPP2024.06.15.599113V3-supplement-1.pdf]
