# Supplementary material for: MPAC: a computational framework for inferring cancer pathway activities from multi-omic data
Source: bioRxiv. 2024 Jun 17:2024.06.15.599113. Preprint. [Version 1] doi: 10.1101/2024.06.15.599113 (PMC11212914; doi:10.1101/2024.06.15.599113)

bioRxiv preprint doi: https://doi.org/10.1101/2024.06.15.599113; this version posted June 17, 2024. The copyright holder for this preprint (which was not certified by peer review) is the author/funder, who has granted bioRxiv a license to display the preprint in perpetuity. It is made available under aCC-BY 4.0 International license.

Supplementary figure legends

Supplementary Figure 1. HPV- exploratory set patient samples grouped by GO term enrichment based on IPL (A), CNA (B), or RNA (C). The selection threshold was lowered to ≥ 3 (**B**) or ≥ 60% (**C**) to include more GO terms to avoid bias.

Supplementary Figure 2. HPV+ exploratory set patient samples grouped by GO term enrichment based on CNA (A) or RNA (B). The selection threshold was lowered to ≥ 2 (**A**) or ≥ 60% (**B**) to include more GO terms to avoid bias.

Supplementary Figure 3. HPV+ exploratory set patient samples grouped by MPAC's input RNA states. (A) Clustering results by K-means under a pre-specified total number of groups ranging from two to six; (**B**) Clustering results by K-means with a pre-specified six groups; (**C–G**) Top significantly enriched GO terms in group I (**C**), II (**D**), III (**E**), IV (**F**), and V (**G**). The selection threshold was lowered to ≥ 95% (**F**), ≥70% (**G**), or ≥50% (**C–E**) in order to include more GO terms to avoid bias.

bioRxiv preprint doi: https://doi.org/10.1101/2024.06.15.599113; this version posted June 17, 2024. The copyright holder for this preprint (which was not certified by peer review) is the author/funder, who has granted bioRxiv a license to display the preprint in perpetuity. It is made available under aCC-BY 4.0 International license.

Supplementary Figure 4. HPV+ exploratory set patient samples grouped by MPAC's input CNA states. (A) Clustering results by K-means under a pre-specified total number of groups ranging from two to six; (**B**) Clustering results by K-means with a pre-specified six groups. Group III, IV, V, and VI contained one sample each.

Supplementary Figure 5. CNA, RNA, and pathway states of CD28 (A), CD86 (B), and LCP2 (C), as well as pathway states of their pathway network neighbors in the eleven group I patients.

Supplementary Figure 6. CNA, RNA, and pathway states of TYK2 (A) and IL12RB1 (B), as well as pathway states of their pathway network neighbors in the eleven group I patients.

Supplementary Figure 7. CNA, RNA, and pathway states of CD247 (A) and FASLG (B), as well as pathway states of their pathway network neighbors in the eleven group I patients.

Supplementary Figure 8. CNA, RNA, and pathway states of FASLG as well as pathway states of its pathway network neighbors in a group I patient TCGA-CR-7385.

Supplementary Figure 9. Overall survival of HPV+ exploratory set patient samples stratified by the inferred pathway levels (IPLs) of proteins from the same submodule (A) or individual protein (B). Not all overall survival tests were statistically significant under a $P < 0.01$ cutoff.

bioRxiv preprint doi: https://doi.org/10.1101/2024.06.15.599113; this version posted June 17, 2024. The copyright holder for this preprint (which was not certified by peer review) is the author/funder, who has granted bioRxiv a license to display the preprint in perpetuity. It is made available under aCC-BY 4.0 International license.

Supplementary Figure 10. Progression-free survival of HPV+ exploratory set patient samples stratified by the inferred pathway levels (IPLs) of all the seven proteins combined (A); proteins from the same submodule (B), or individual protein (C). Not all progression-free survival tests were statistically significant under a P < 0.01 cutoff.

Supplementary Figure 11. Immune cell compositions stratified by the inferred pathway levels (IPLs) of all the seven proteins combined for HPV+ exploratory (A) and validation (B) set. Adjusted Wilcoxon test P on the cell composition difference between two groups of patient samples were shown for each cell type. Eosinophil was not found in either the exploratory or validation sets. T cell CD4+ naïve was not found in the validation set.

Supplementary Figure 12. Overall survival of HPV+ validation set patient samples stratified by the inferred pathway levels (IPLs) of proteins from the same submodule (A) or individual protein (B). Not all overall survival tests were statistically significant under a P < 0.01 cutoff.

bioRxiv preprint doi: https://doi.org/10.1101/2024.06.15.599113; this version posted June 17, 2024. The copyright holder for this preprint (which was not certified by peer review) is the author/funder, who has granted bioRxiv a license to display the preprint in perpetuity. It is made available under aCC-BY 4.0 International license.

Supplementary Figure 1

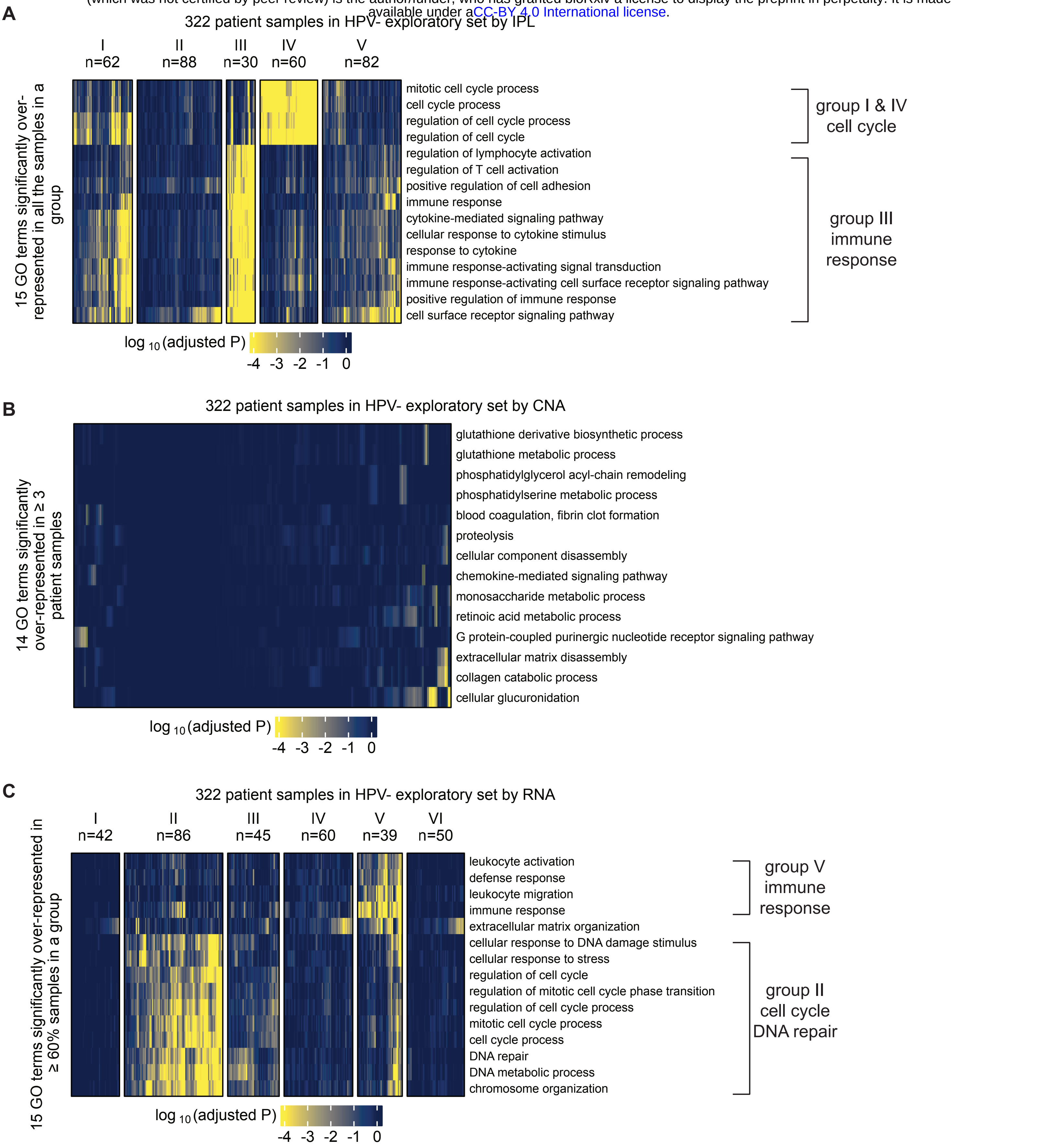

bioRxiv preprint doi: https://doi.org/10.1101/2024.06.15.599113; this version posted June 17, 2024. The copyright holder for this preprint (which was not certified by peer review) is the author/funder, who has granted bioRxiv a license to display the preprint in perpetuity. It is made available under aCC-BY 4.0 International license.

Supplementary Figure 2

A

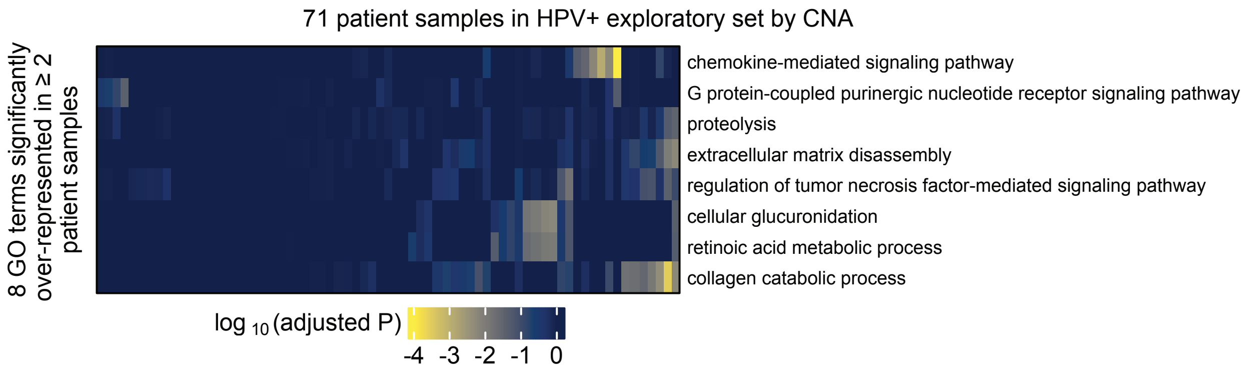

B

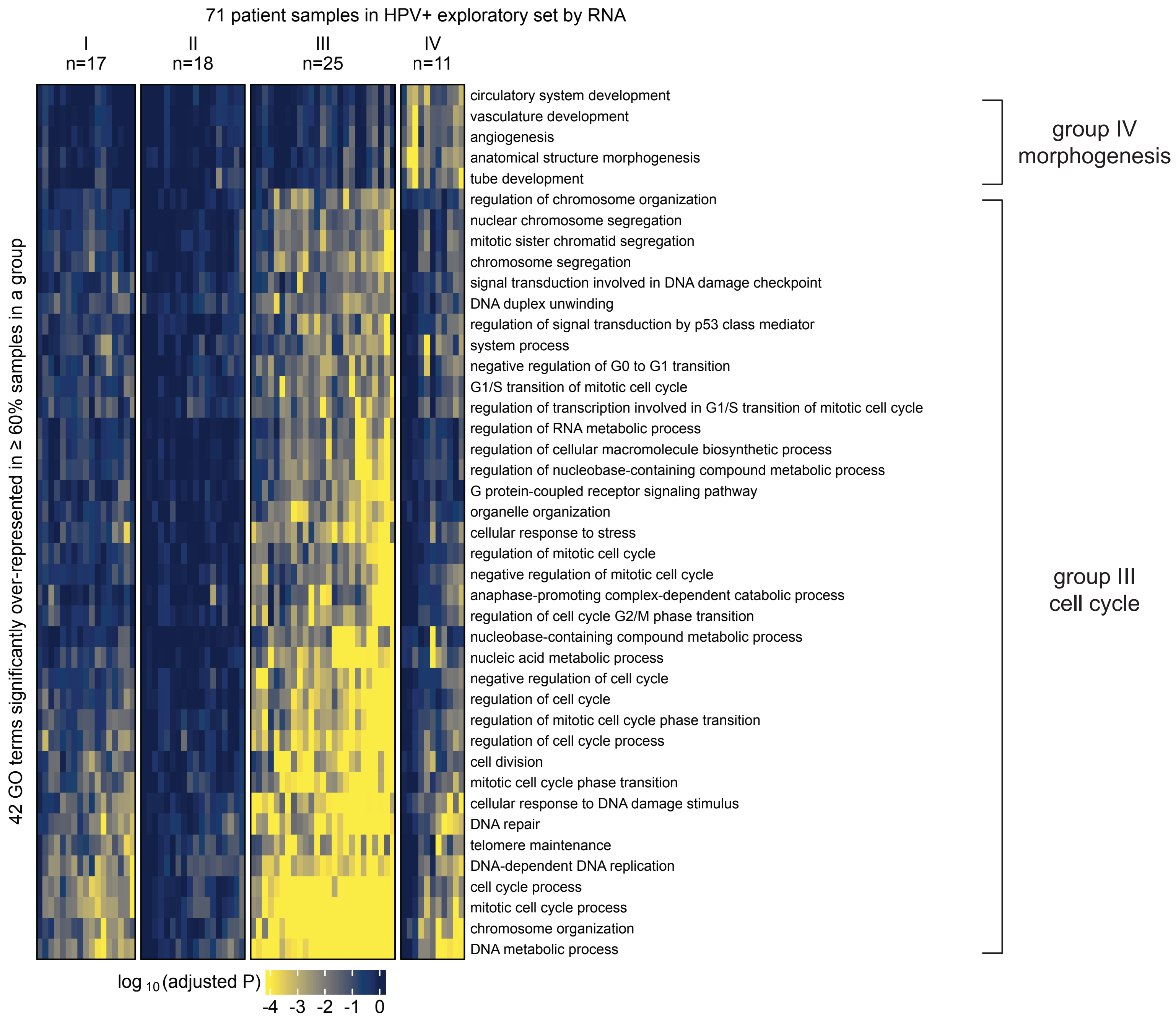

Supplemenary Figure 3

bioRxiv preprint doi: https://doi.org/10.1101/2024.06.15.599113; this version posted June 17, 2024. The copyright holder for this preprint (which was not certified by peer review) is the author/funder, who has granted bioRxiv a license to display the preprint in perpetuity. It is made available under aCC-BY 4.0 International license.

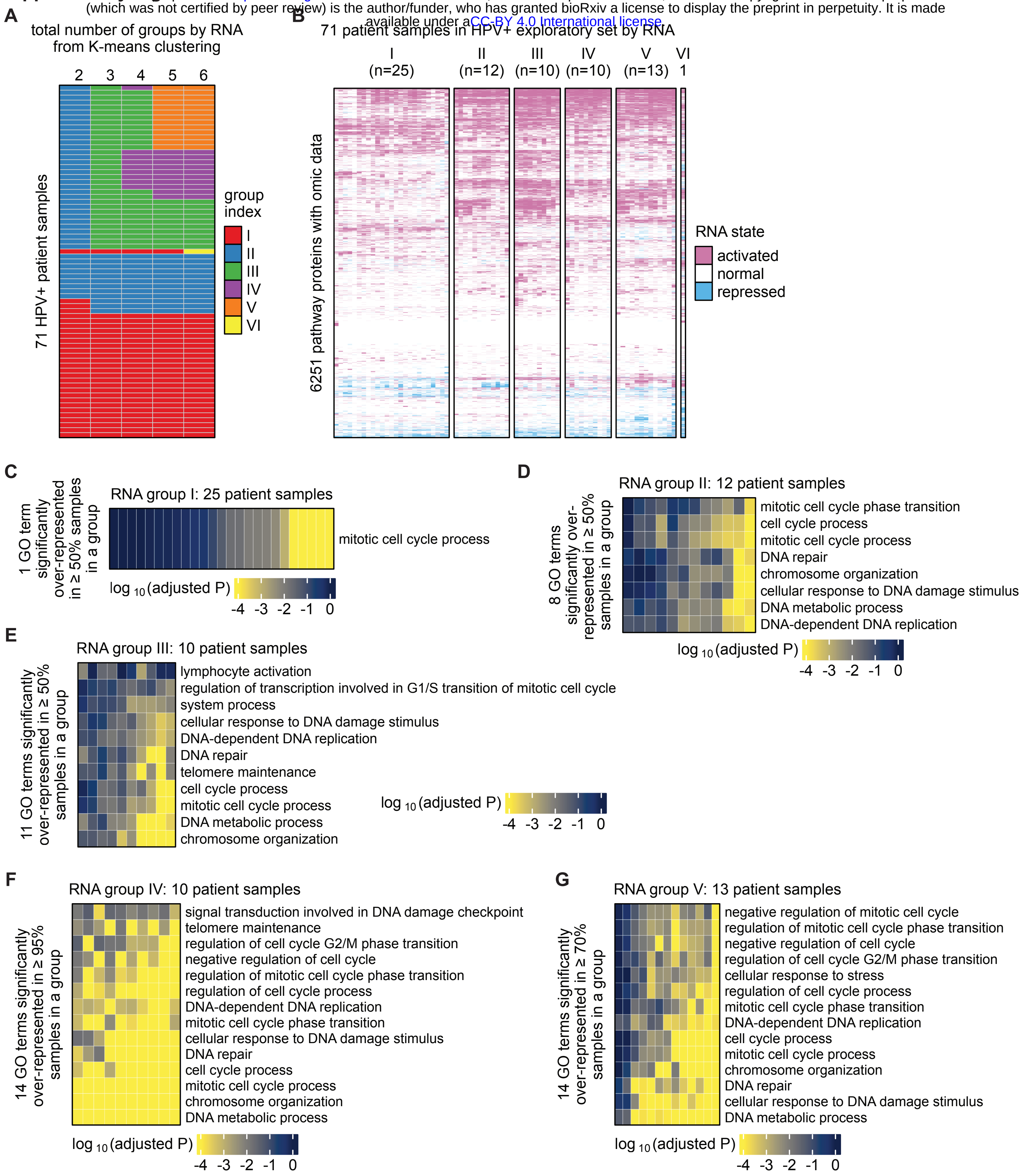

bioRxiv preprint doi: https://doi.org/10.1101/2024.06.15.599113; this version posted June 17, 2024. The copyright holder for this preprint (which was not certified by peer review) is the author/funder, who has granted bioRxiv a license to display the preprint in perpetuity. It is made available under aCC-BY 4.0 International license.

Supplementary Figure 4

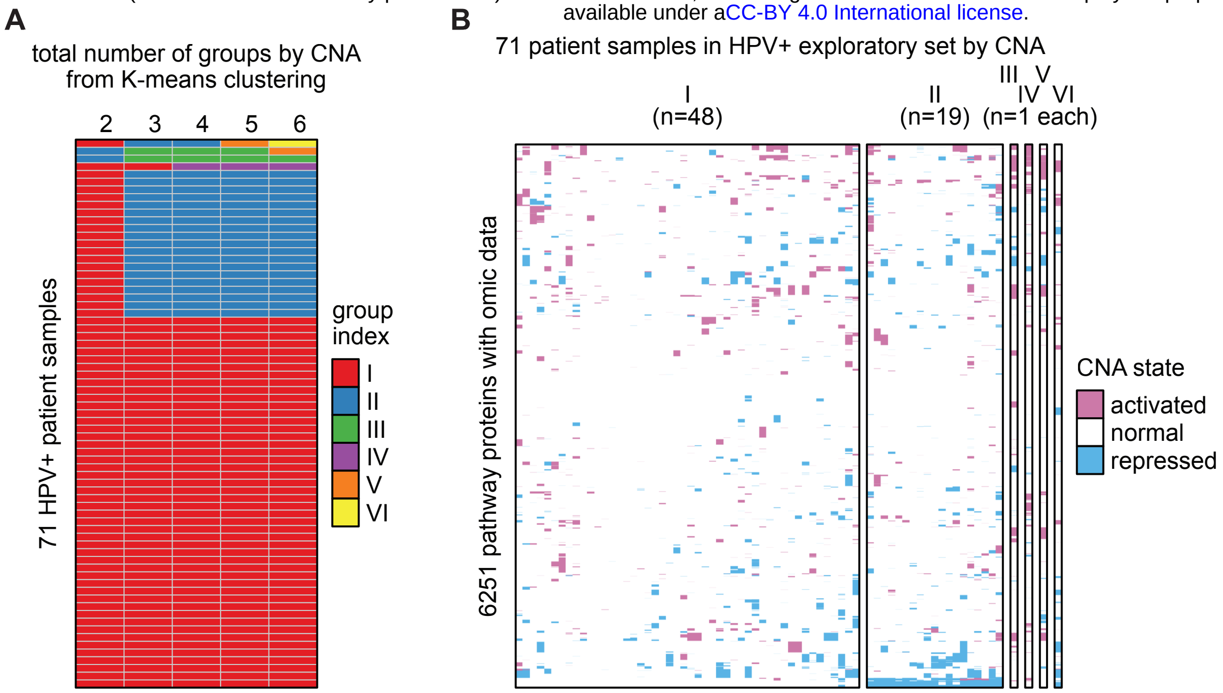

bioRxiv preprint doi: https://doi.org/10.1101/2024.06.15.599113; this version posted June 17, 2024. The copyright holder for this preprint (which was not certified by peer review) is the author/funder, who has granted bioRxiv a license to display the preprint in perpetuity. It is made available under aCC-BY 4.0 International license.

Supplementary Figure 5

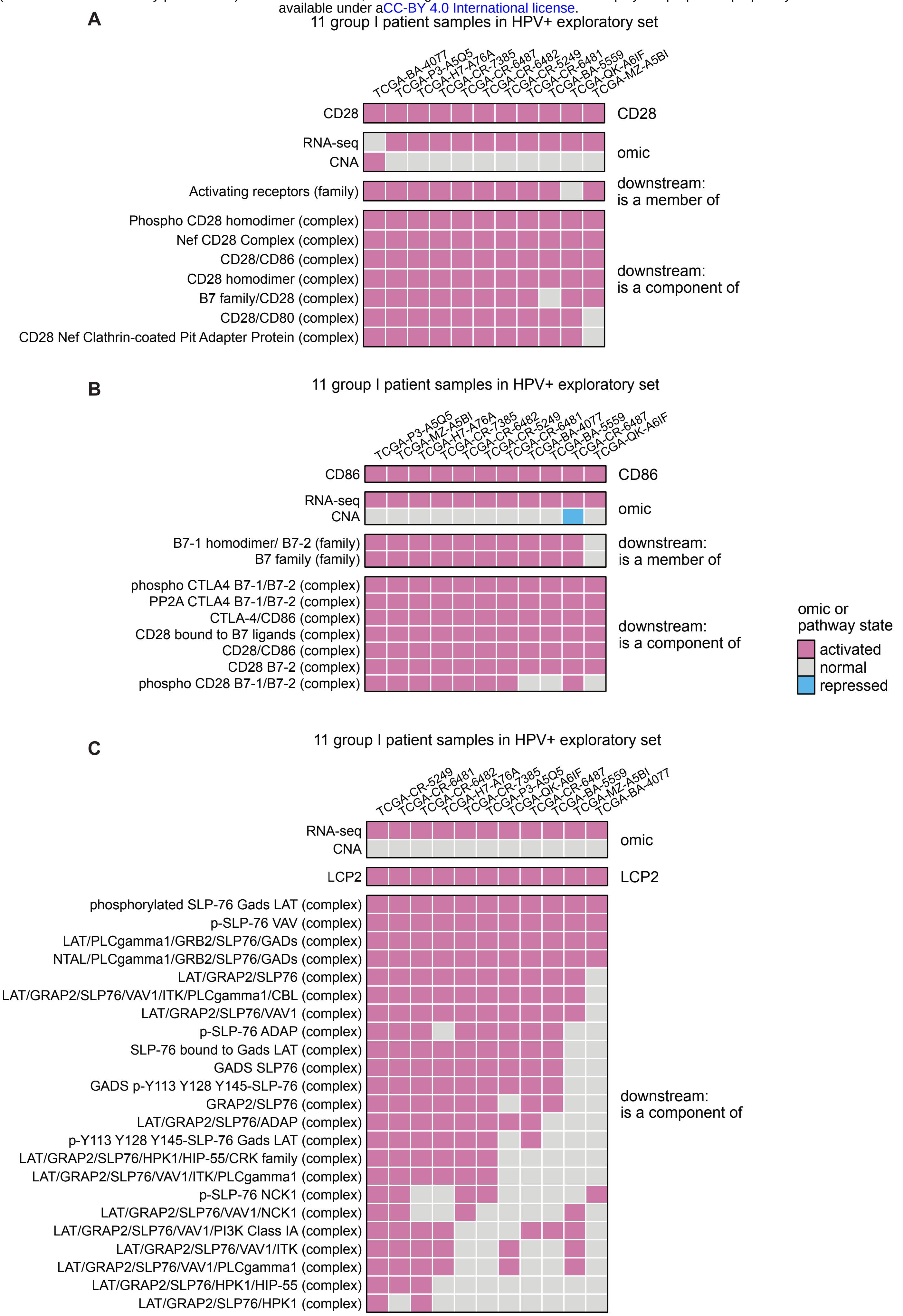

bioRxiv preprint doi: https://doi.org/10.1101/2024.06.15.599113; this version posted June 17, 2024. The copyright holder for this preprint (which was not certified by peer review) is the author/funder, who has granted bioRxiv a license to display the preprint in perpetuity. It is made available under aCC-BY 4.0 International license.

Supplemental Figure 6

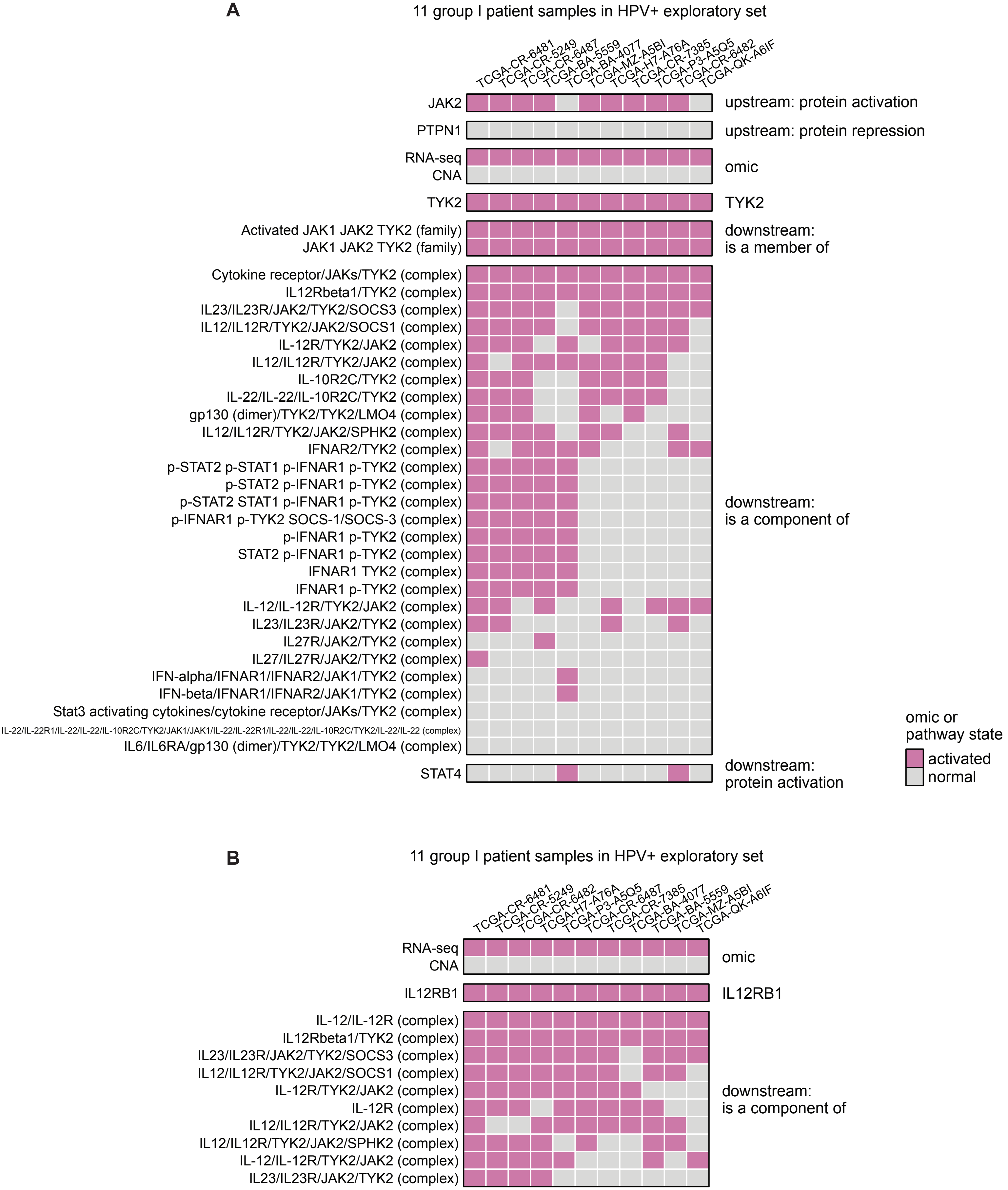

Supplementary Figure 7

bioRxiv preprint doi: https://doi.org/10.1101/2024.06.15.599113; this version posted June 17, 2024. The copyright holder for this preprint (which was not certified by peer review) is the author/funder, who has granted bioRxiv a license to display the preprint in perpetuity. It is made available under aCC-BY 4.0 International license.

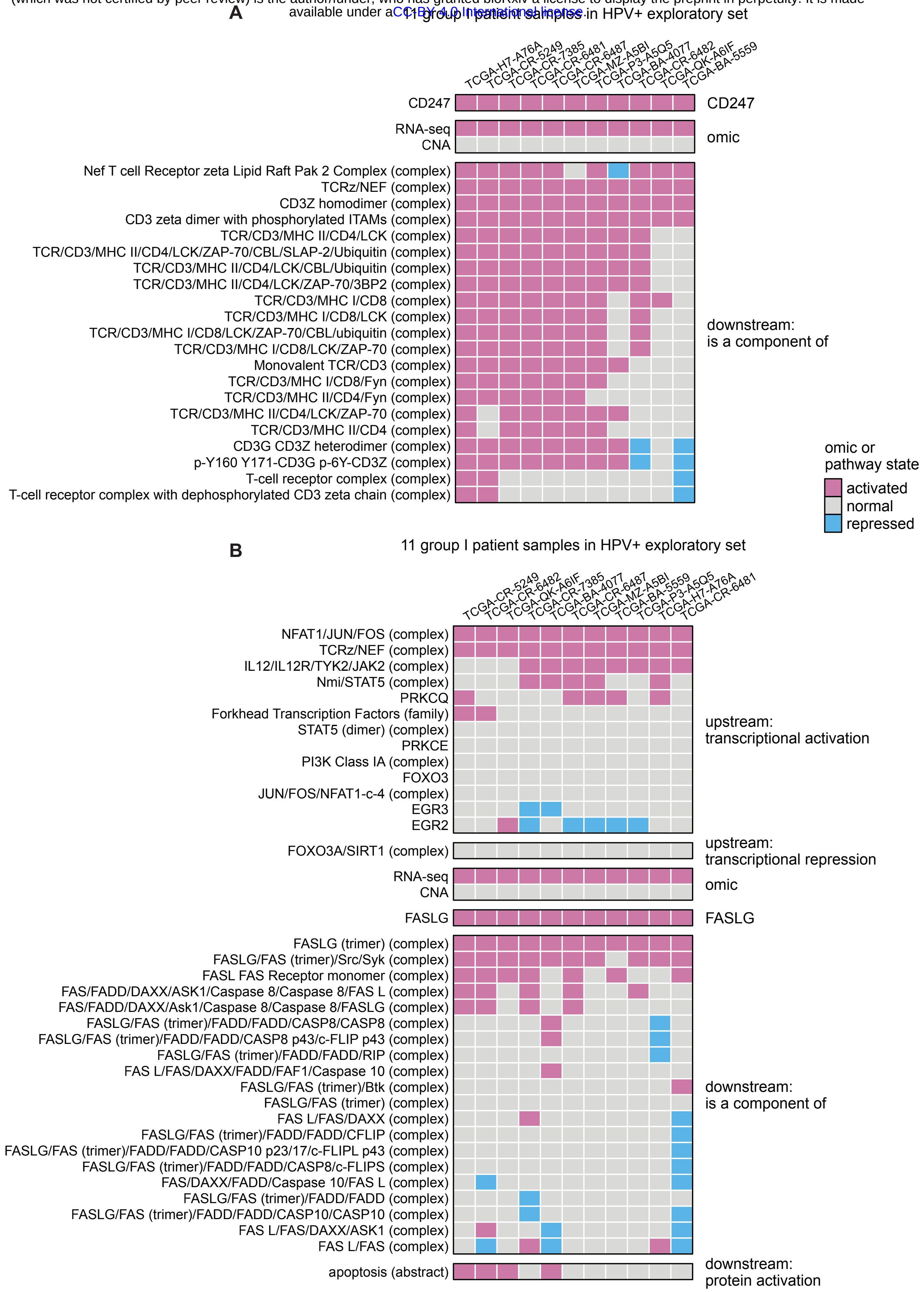

Supplementary Figure 8

bioRxiv preprint doi: https://doi.org/10.1101/2024.06.15.599113; this version posted June 17, 2024. The copyright holder for this preprint (which was not certified by peer review) is the author/funder, who has granted bioRxiv a license to display the preprint in perpetuity. It is made available under aCC-BY 4.0 International license.

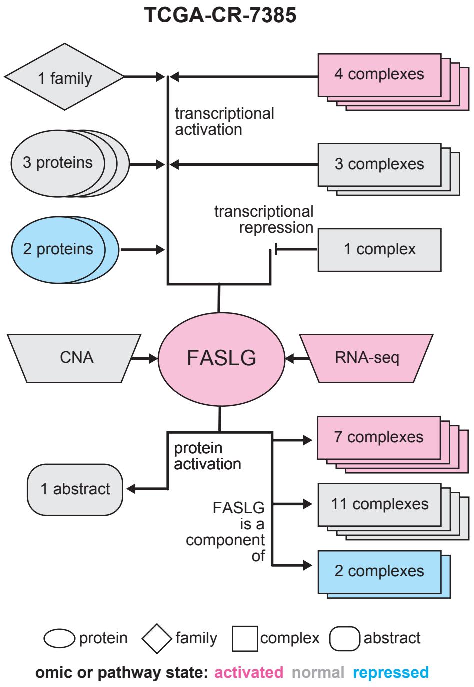

bioRxiv preprint doi: https://doi.org/10.1101/2024.06.15.599113; this version posted June 17, 2024. The copyright holder for this preprint (which was not certified by peer review) is the author/funder, who has granted bioRxiv a license to display the preprint in perpetuity. It is made available under aCC-BY 4.0 International license.

Supplementary Figure 9

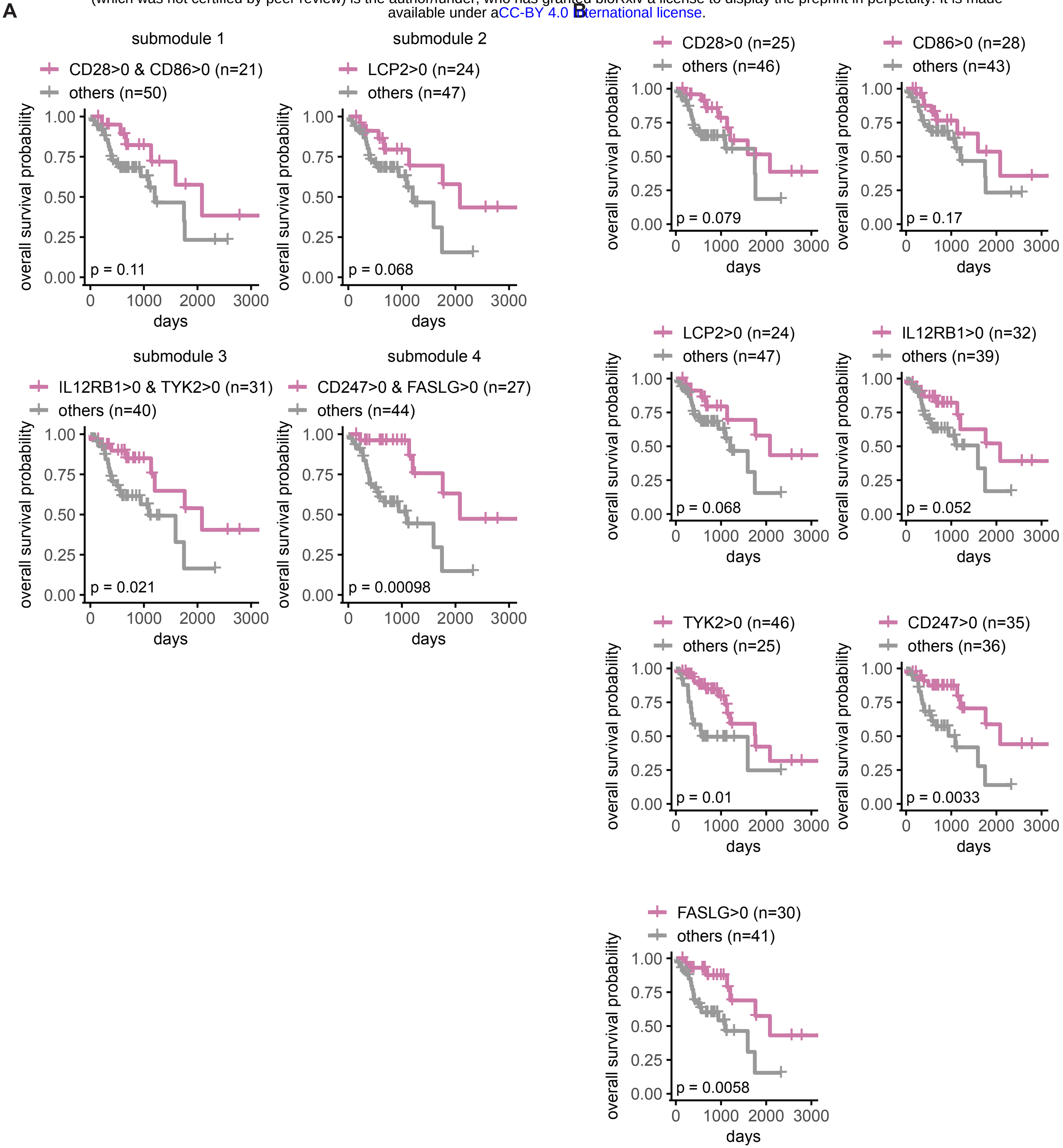

bioRxiv preprint doi: https://doi.org/10.1101/2024.06.15.599113; this version posted June 17, 2024. The copyright holder for this preprint (which was not certified by peer review) is the author/funder, who has granted bioRxiv a license to display the preprint in perpetuity. It is made available under aCC-BY 4.0 International license.

Supplementary Figure 10

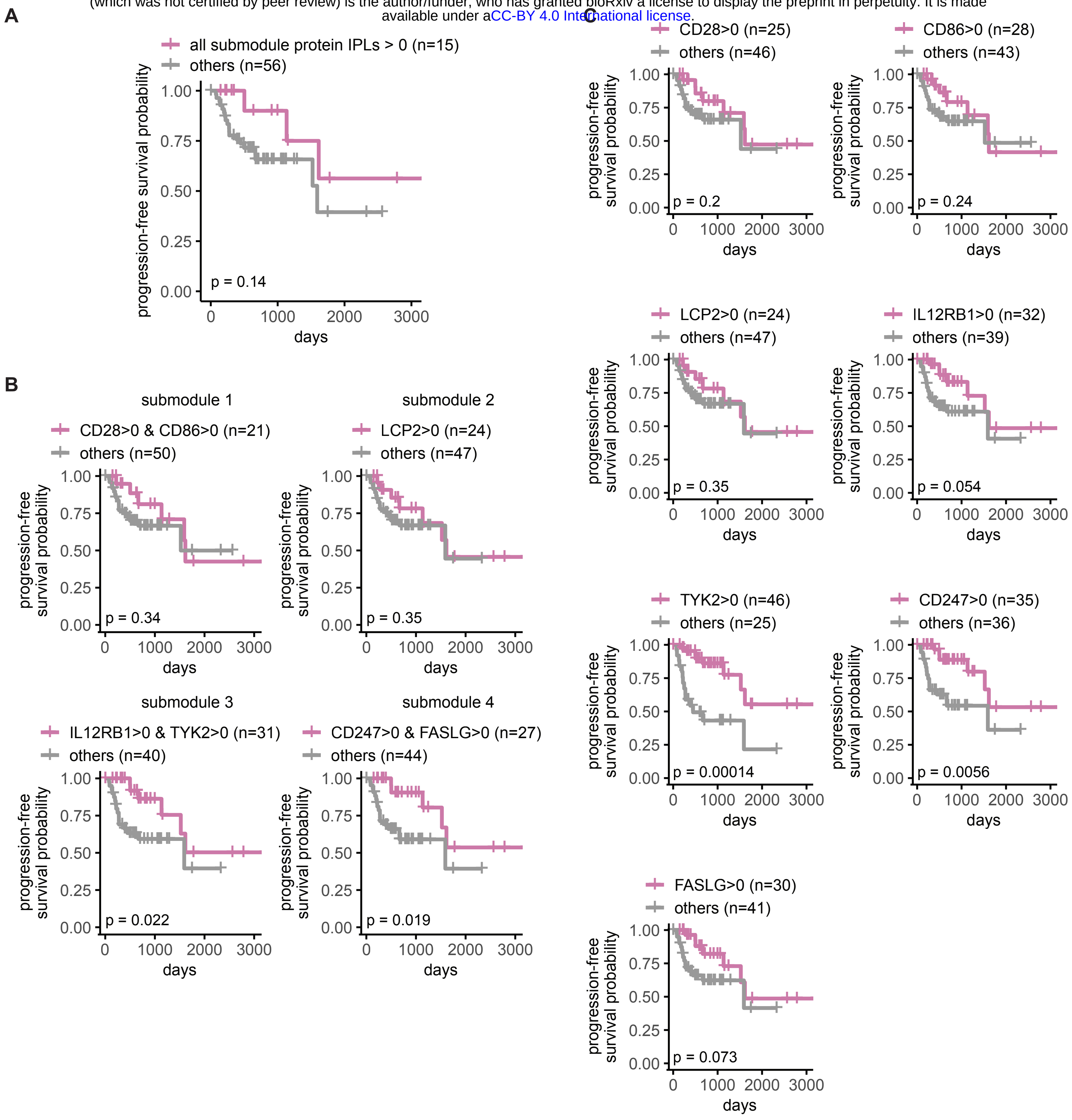

Supplementary Figure 11

bioRxiv preprint doi: https://doi.org/10.1101/2024.06.15.599113; this version posted June 17, 2024. The copyright holder for this preprint (which was not certified by peer review) is the author/funder, who has granted bioRxiv a license to display the preprint in perpetuity. It is made available under aCC-BY 4.0 International license.

A

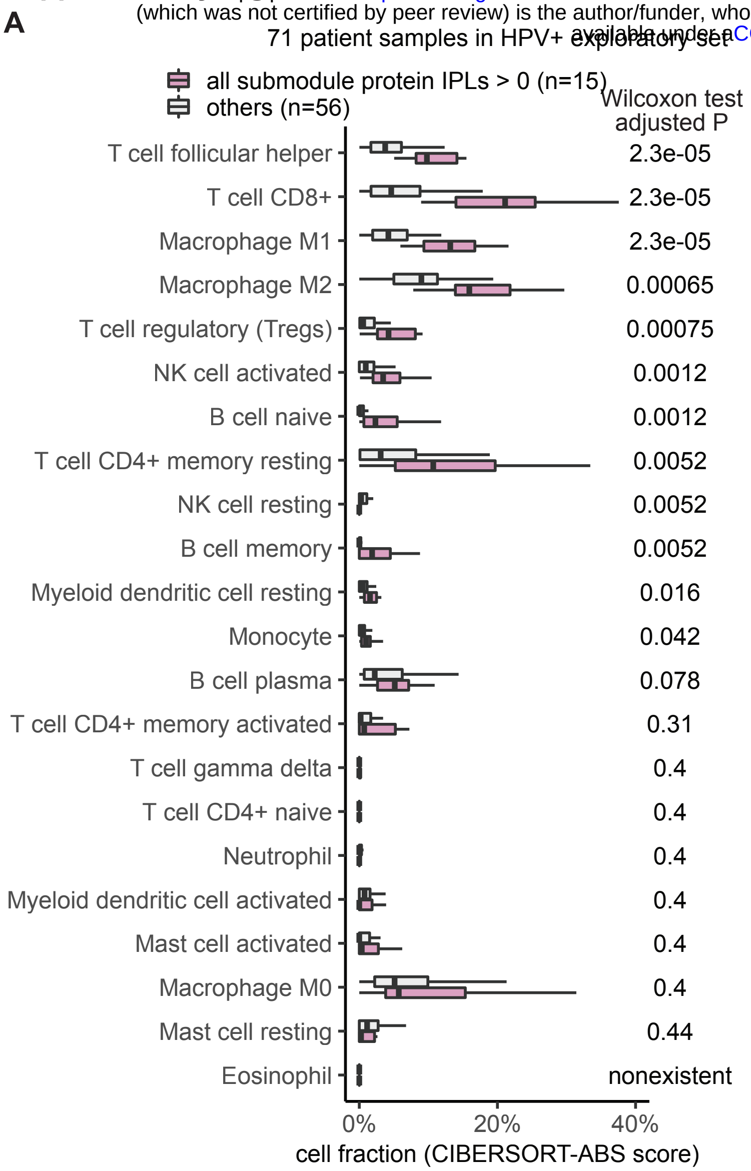

B

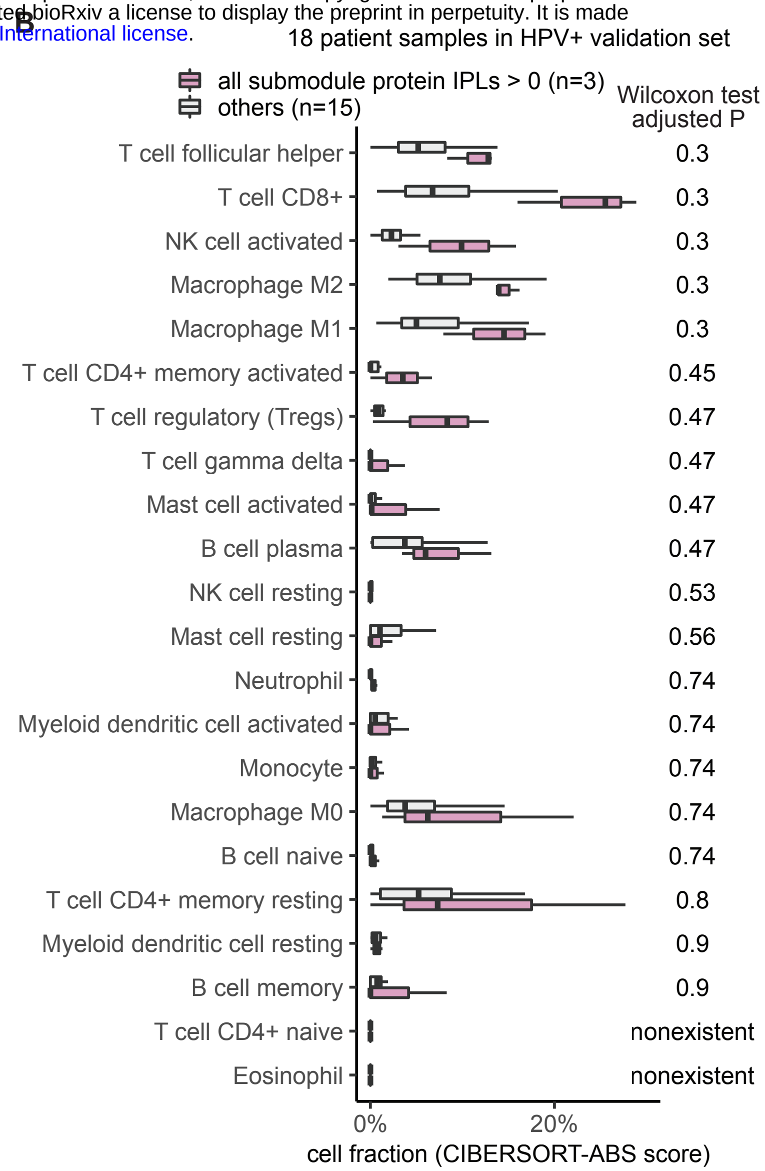

Supplementary Figure 12

bioRxiv preprint doi: https://doi.org/10.1101/2024.06.15.599113; this version posted June 17, 2024. The copyright holder for this preprint (which was not certified by peer review) is the author/funder, who has granted bioRxiv a license to display the preprint in perpetuity. It is made available under aCC-BY 4.0 International license.

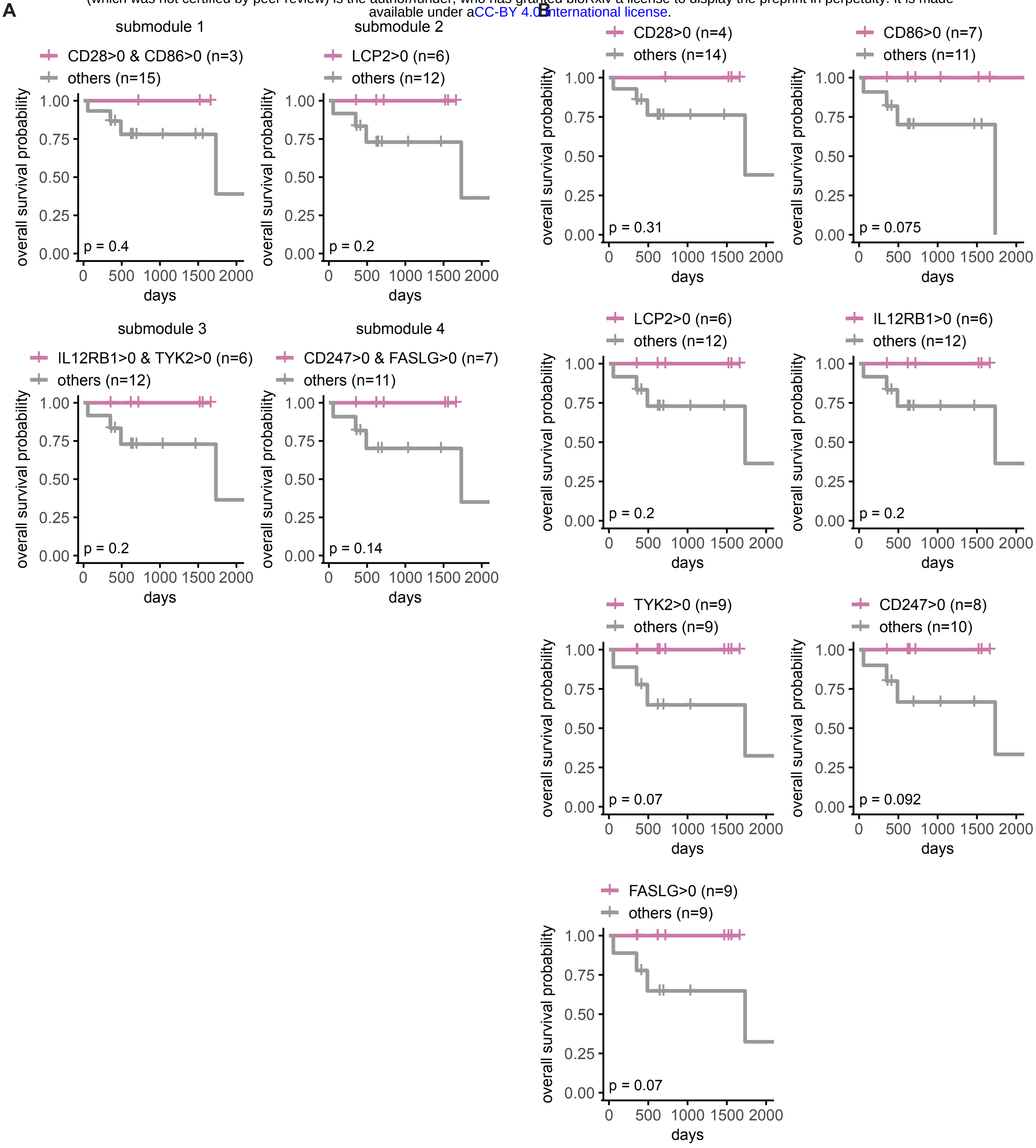

Supplement: Supplementary file 1 [file NIHPP2024.06.15.599113v1-supplement-1.pdf]
